# Discovery of multiple anti-CRISPRs highlights anti-defense gene clustering in mobile genetic elements

**DOI:** 10.1038/s41467-020-19415-3

**Published:** 2020-11-06

**Authors:** Rafael Pinilla-Redondo, Saadlee Shehreen, Nicole D. Marino, Robert D. Fagerlund, Chris M. Brown, Søren J. Sørensen, Peter C. Fineran, Joseph Bondy-Denomy

**Affiliations:** 1 Section of Microbiology, University of Copenhagen, Copenhagen, Denmark; 2 Department of Microbiology and Immunology, University of California, San Francisco, CA USA; 3 University College Copenhagen, Copenhagen, Denmark; 4 Department of Biochemistry, University of Otago, Dunedin, New Zealand; 5 Department of Microbiology and Immunology, University of Otago, Dunedin, New Zealand; 6 Genetics Otago, University of Otago, Dunedin, New Zealand; 7 Bio-protection Research Centre, University of Otago, Dunedin, New Zealand; 8 Quantitative Biosciences Institute, UCSF, San Francisco, CA USA; 9 Innovative Genomics Institute, Berkeley, CA USA

**Keywords:** Bacteria, CRISPR-Cas systems

## Abstract

Many prokaryotes employ CRISPR–Cas systems to combat invading mobile genetic elements (MGEs). In response, some MGEs have developed strategies to bypass immunity, including anti-CRISPR (Acr) proteins; yet the diversity, distribution and spectrum of activity of this immune evasion strategy remain largely unknown. Here, we report the discovery of new Acrs by assaying candidate genes adjacent to a conserved Acr-associated (Aca) gene, *aca5*, against a panel of six type I systems: I–F (*Pseudomonas*, *Pectobacterium*, and *Serratia*), I–E (*Pseudomonas* and *Serratia*), and I–C (*Pseudomonas*). We uncover 11 type I–F and/or I–E anti-CRISPR genes encoded on chromosomal and extrachromosomal MGEs within *Enterobacteriaceae* and *Pseudomonas*, and an additional Aca (*aca9*). The *acr* genes not only associate with other *acr* genes, but also with genes encoding inhibitors of distinct bacterial defense systems. Thus, our findings highlight the potential exploitation of *acr* loci neighborhoods for the identification of previously undescribed anti-defense systems.

## Abstract (web summary)

Mobile genetic elements have evolved anti-CRISPR (Acr) proteins to bypass the immunity provided by prokaryotic CRISPR–Cas systems. Here, the authors identify 11 type I Acrs encoded on mobile genetic elements, and show that *acr* loci neighborhoods can be used to discover inhibitors of other bacterial defense systems.

## Introduction

All cellular life is under the constant threat of invasion by foreign genetic elements. Prokaryotes are outnumbered by a wide spectrum of mobile genetic elements (MGEs) that infect them, including viruses and plasmids. This selective pressure has driven the evolution of diverse defense mechanisms, including restriction-modification systems, abortive infection, and clustered regularly interspaced short palindromic repeats (CRISPR) and CRISPR-associated (Cas) genes^1,2^.

CRISPR–Cas loci have been identified in sequenced genomes of around 40% of bacteria and 85% of archaea^3^ and are occasionally carried by a wide range of MGEs^4–6^, bearing testament to their evolutionary and ecological importance. This mode of defense allows cells to remember, recognize and thwart recurrently infecting agents. Broadly, CRISPR–Cas immunity consists of three main stages: adaptation, processing/biogenesis, and interference^7^. During adaptation, snippets of an invading genetic element are incorporated into CRISPR arrays as “spacers” between repeat sequences, yielding a heritable record of former genetic intruders. The CRISPR array is then expressed as a long transcript (pre-crRNA) that is processed into single CRISPR RNAs (crRNAs), which guide Cas nucleases to target invading nucleic acids that carry a complementary sequence to the spacer (referred to as protospacer).

In response to the strong selective pressure exerted by CRISPR–Cas immunity, many MGEs have developed inhibitors of CRISPR–Cas function called anti-CRISPR (Acr) proteins^8^. The first *acr* genes were discovered in phages that inhibit the type I–F CRISPR–Cas system of *Pseudomonas aeruginosa*^9^. Many more non-homologous Acr proteins have been subsequently reported for different CRISPR–Cas types (e.g., types II, III, and V)^10–15^, including some on non-phage MGEs^16^. Previous structural and biochemical characterization of Acr proteins has revealed a diverse range of inhibitory activities, including interference with crRNA loading, inhibition of target DNA recognition, and inhibition of DNA cleavage, among others^17^. Apart from sharing a typically low molecular weight, Acrs lack conserved sequence and structural features, thus rendering de novo prediction largely impractical with current methods. However, *acr* genes tend to cluster within loci that encode more conserved Acr-associated (Aca) proteins^8,18^, which are transcriptional repressors of the *acr* locus^19,20^. The *aca* genes are often more broadly distributed than *acr* genes and have been used to uncover new *acr* loci^12,14,18,21^.

The discovery of Acr proteins explains how MGEs can persist despite frequent targeting by host spacer sequences. Acrs are predicted to be a strong driver for the diversification of CRISPR–Cas systems in nature and the accumulation of other defense systems in prokaryotic genomes. Because MGEs facilitate host genome rearrangements and provide the foundation for vast prokaryotic gene exchange networks, the study of Acrs allows a better understanding of MGE-host interactions and the horizontal transfer potential of MGE-encoded traits (e.g., antibiotic resistance) across microbiomes. Practically, Acr proteins also benefit phage-based therapeutics and plasmid-based delivery platforms and provide a means to control CRISPR–Cas-derived biotechnologies^22^.

In this study, we investigate the interactions between MGEs and their bacterial hosts, focusing on uncovering new Acrs that enable MGEs to avoid potent host defense mechanisms. We describe the discovery of 11 type I–F and/or I–E Acr families encoded by phage and non-phage MGEs by leveraging *aca5* and a newly identified *aca* gene (*aca9*) as markers for *acr* loci. Bioinformatic analyses further revealed that *acrs* co-locate with other anti-defense systems within MGE genomes, suggesting the existence of anti-defense gene clusters and highlighting a potential avenue for the discovery of unknown anti-defense genes.

## Results

### An *aca5*-based computational search for Acr candidates

**Fig. 1:**
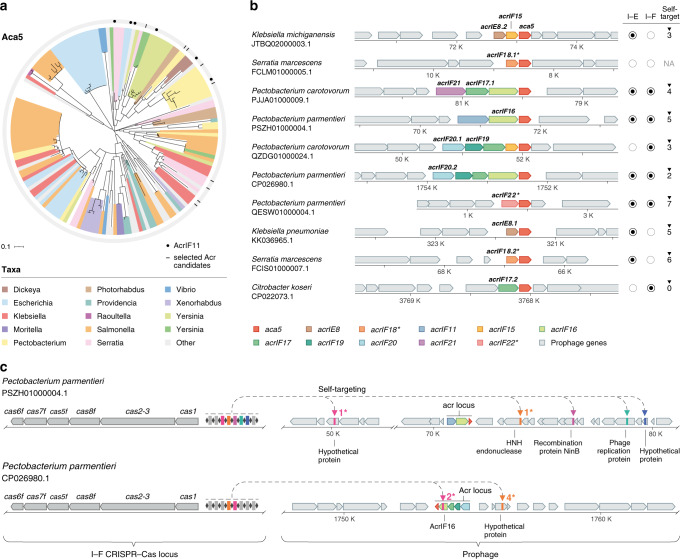
Bioinformatic search of *aca5*-associated *acrs*. **a** Phylogenetic distribution of Aca5 homologs across bacterial taxa. Tree branches are color-coded according to the genus from which the Aca5 orthologs originate. Black circles in the outer ring depict instances where Aca5 is associated with AcrIF11, marking the starting points for the guilt-by-association search; black lines represent the genomes from which *acr* candidates were selected for functional testing. **b** Genomic organization of the *acrs* selected for testing. Genes are colored by Acr family and the surrounding prophage genomic contexts are depicted in gray. Specific orthologs selected for testing are highlighted in bold. The presence/absence of I–F and I–E CRISPR–Cas systems and self-targeting spacers in the host genomes are summarized on the right (for details, see Supplementary Data 1 and 2). Self-targeting analysis was not possible in genomes where CRISPR–Cas systems were not detected, marked here as “NA”. **c** Graphic representation of the self-targeting instances observed in two *Pectobacterium parmentieri* genomes from which *acrIF16* and *acrIF20* genes were selected for testing. Colored arrowheads indicate the positions within the prophages that are targeted by a spacer in one of the host’s I–F CRISPR arrays. Genes are colored according to the legend in (**b**). When possible, the name of the targeted gene is provided. All protospacers were flanked by the I–F 5’-GG-3’ PAM (Supplementary Data 2). Asterisks next to arrowheads indicate the number of spacer-protospacer mismatches; Two out of the seven protospacers were found to have a mismatch within the seed region (Supplementary Fig. 1).

To uncover how MGEs within the Enterobacteriales order cope with the pressure of CRISPR–Cas immunity, we performed bioinformatic searches using the Enterobacteriales-enriched *aca5* gene^12^ that is encoded downstream of several *acrIF11* orthologs. These searches revealed a wide phylogenetic distribution of *aca5* homologs across bacterial families (Fig. 1a), including members of the *Salmonella, Pectobacterium, Klebsiella, Serratia*, and *Escherichia* genera. Distant homologs were also identified in other bacterial orders (e.g., Vibrionales) at a considerably lower prevalence (<5%). Importantly, these organisms are enriched with class 1 CRISPR–Cas systems (primarily types I–F and/or I–E)^3^, suggesting that their MGEs may rely on unknown type I inhibitors to bypass immunity.

Based on common characteristics of known Acr proteins, we restricted our candidate list to small predicted proteins (<200 amino acids) encoded upstream of *aca5* within genomic regions containing numerous MGE-associated genes. Following this approach, we identified several *acr* candidates residing in prophages from genomes of *Pectobacterium*, *Serratia*, *Klebsiella* and *Citrobacter*, and 10 genomes harboring a diverse set of putative *acr* genes were selected for further study (Fig. 1b and Supplementary Data 1). Notably, while the position of *aca5* remained fixed across these putative *acr* operons, *acr* candidates often co-occurred in shuffled clusters of 2–4 genes, as seen for other *acr* loci^9,12,13,23^.

Bacteria that express *acrs* can tolerate “self-targeting” spacers that, in the absence of CRISPR–Cas inhibition, would otherwise cause lethal genomic cleavage^9,24–26^. The presence of these self-targeting spacers can therefore be used to identify bacterial genomes that likely encode anti-CRISPR proteins capable of inhibiting their endogenous CRISPR system^12,13,15,27^. We found that 8 out of the 10 selected genomes contained several I–F and/or I–E self-targeting spacers (Fig. 1b, right and Supplementary Data 1 and 2). A large number of these spacers (21/34—62% of all self-targeting hits) matched targets within the predicted prophages carrying the *acr* candidates (Supplementary Data 2). Due to the promiscuous PAM of I–E^28–30^, we were unable to confidently ascertain whether the PAM would enable targeting of the predicted spacer-protospacer matches. However, most I–F protospacers (83%) were flanked by the conserved I–F 5’-GG-3’ PAM, as described previously^31,32^ (Fig. 1c and Supplementary Data 2). Collectively, we concluded that the identified prophage genomes likely harbored *acr* loci and proceeded to experimentally test the 9 selected type I–E/I–F candidate *acr* genes.

### Newly identified Acr proteins inhibit different type I CRISPR–Cas systems

**Fig. 2:**
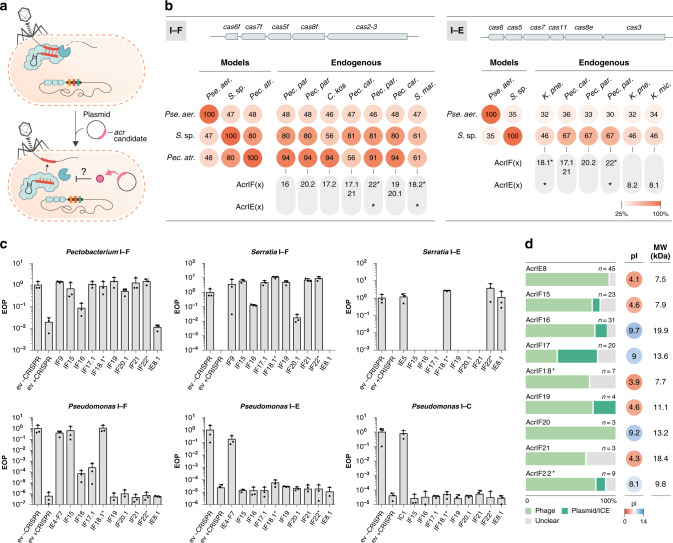
Newly identified *acr* genes inhibit diverse type I CRISPR–Cas systems. **a** Schematic of the experimental setup employed for *acr* validation. Bacteria carrying their native type I–F, I–E, or I–C CRISPR–Cas systems and a plasmid expressing each *acr* candidate were challenged with a CRISPR-targeted phage and infectivity was assessed. **b** Percent sequence identity comparison between the model CRISPR–Cas systems challenged (type I–F: *Pectobacterium*, *Serratia*, and *Pseudomonas*; type I–E: *Serratia* and *Pseudomonas;* type I–C: *Pseudomonas*) and the type I–F/I–E systems found in the endogenous hosts of the *acr* candidates, when available. Average values for the individual percentage identity comparisons between Cas orthologs forming the type I–E/I–F interference complexes are shown, excluding the adaptation modules (see top gene map) (Supplementary Data 3). The specific Acr orthologs found in the genomes which were selected for testing are noted below; asterisks indicate dual anti-I–F/I–E function. **c** Efficiency of plaquing (EOP) of CRISPR-targeted phages in bacterial lawns expressing the different *acr* candidates, or the empty vector control (ev +CRISPR), compared to EOP of the same phage in non-targeting bacterial lawns carrying the empty vector (ev −CRISPR). Positive controls for Acr inhibition include AcrIF9 (*Pectobacterium* and *Serratia* I–F), AcrIE5 (*Serratia* I–E), AcrIE4-F7 (*Pseudomonas* I–F and I–E), and AcrIC1 (*Pseudomonas* I–C). Data are presented as mean ± SEM (*n* = 3 biologically independent samples). In *Serratia*, CRISPR immunity provided an EOP of ~1 × 10^−6^ and the absence of EOP data indicates that no single plaques were detected with ~1 × 10^9^ pfu mL^−1^ of phage JS26. **d** Percentage distribution of the MGE origin (phage, plasmid/ICE, or unclear) for the collection of orthologs of each of the validated *acrs*. The isoelectric point (pI) and molecular weights (MW) of the validated *acrs* are shown. Source data are available in the Source data file.

The nine candidate *acr* genes (and four additional homologs, denoted here by a decimal number after the Acr name) were tested against a panel of type I CRISPR–Cas systems: two type I–E variants (*Serratia* sp. ATCC39006 and *Pseudomonas aeruginosa* SMC4386), three type I–F variants (*Serratia* sp. ATCC39006, *Pseudomonas aeruginosa* PA14, and *Pectobacterium atrosepticum* SCRI1043), and one type I–C system (engineered strain of *Pseudomonas aeruginosa* PAO1). We challenged each of the model organisms with a CRISPR-targeted phage and assayed whether its replication could be restored by an *acr* candidate gene (Fig. 2a). Notably, the different CRISPR–Cas subtypes and variants in the chosen model organisms span a wide phylogenetic diversity. While some of the tested systems display high degrees of similarity (e.g., >80% a.a. identity) with the CRISPR–Cas systems present in the endogenous *acr* hosts, others exhibit high divergence (<50% a.a. identity) (Fig. 2 and Supplementary Data 3), allowing us to test for possible differences in the breadth of inhibitory activity.

Our screening revealed that each of the 9 candidate *acr* families tested inhibited the function of either one or more of the CRISPR–Cas subtype/variants challenged (Fig. 2c). Most candidates (6/9) potently inhibited the *Pectobacterium* and *Serratia* type I–F systems; only a few inhibited the *Serratia* type I–E and/or *Pseudomonas* type I–F strongly (3/9 and 2/9, respectively), and none affected the function of the *Pseudomonas* type I–E and I–C systems. Overall, these results are consistent with the higher similarities between the type I–F and I–E CRISPR–Cas system variants present in the hosts encoding the *acrs* and the *Pectobacterium* and *Serratia* model system variants used for testing (Fig. 2b). Interestingly, our results revealed that AcrIF18.1* and AcrIF22* exhibit broad inhibitory functions, robustly inhibiting the *Serratia* type I–E system and diverse type I–F variants (dual subtype inhibition denoted with an asterisk) (Fig. 2b, c). In addition to AcrIF18.1*, AcrIF15 strongly inhibited all three type I–F systems, whereas AcrIF16 and AcrIF17.1 inhibited *Pseudomonas* type I–F immunity less potently in our assays. AcrIE8 was the only type I–E-specific inhibitor identified in our work. All of the *acr* homologs tested (AcrIF20.2—65% identity, AcrIE8.2—76%, AcrIF18.2*—96%, and AcrIF17.2—39%) showed comparable inhibitory activities to their counterparts, with the exception of AcrIF17.2 from *Citrobacter* which, unlike its distant homolog in *Pectobacterium*, did not inhibit the type I–F system from *P. aeruginosa* (Supplementary Fig. 2). Our results indicate that Acrs tend to strongly inhibit the CRISPR-Cas subtype variants that are closely related to the one encoded by their host bacteria, yet certain Acr proteins (e.g. AcrIF15 and AcrIF18*) show remarkable inhibitory breadth, robustly blocking I-F CRISPR-Cas variants displaying <50 % amino acid identity (Fig. 2b, c).

### Identified Acrs are spread across diverse Proteobacteria and MGE types

To explore the phylogenetic distribution of the new Acrs, publicly available prokaryotic sequences at NCBI were searched for homologs. The resulting analyses revealed a heterogeneous Acr distribution across Proteobacteria, most belonging to *Enterobacteriaceae* (Supplementary Fig. 3 and Supplementary Data 4). As expected, the total collection of hosts encoding these *acrs* showed enrichment of genera frequently encoding CRISPR–Cas types I–F and/or I–E (e.g., *Salmonella*, *Serratia*, *Cronobacter*, *Klebsiella*, *Pectobacterium*) (Supplementary Data 1). Interestingly, while AcrIF19–22 are primarily confined to species of the *Pectobacterium* genus, the rest (AcrIF15–IF18* and AcrIE8) radiate over wider phylogenetic ranges (Supplementary Fig. 3 and Supplementary Data 4). For instance, homologs of AcrIF17 and AcrIF16 are present in bacterial families in addition to *Enterobacteriaceae*, such as *Pseudomonadaceae*, *Burkholderiaceae*, *Halomonadaceae*, and *Desulfobacteraceae*, and *Yersiniaceae*, *Vibrionaceae*, and *Shewanellaceae*, respectively.

**Fig. 3:**
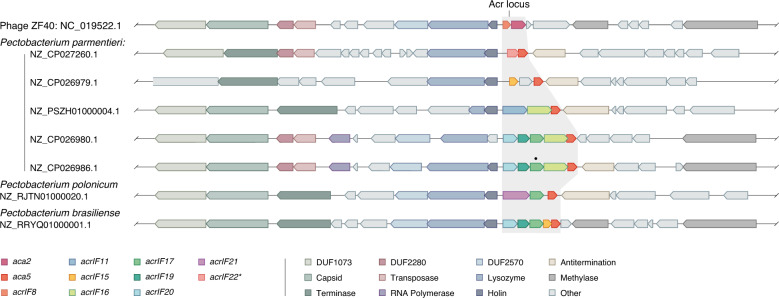
Genomic comparison between *Pectobacterium* phage ZF40 and different *Pectobacterium* genomes with related prophage regions. The alignment of the *acr* loci in related prophages/MGEs shows variability in nearby genes. Genes are color-coded according to their functional annotations. Gene annotations are based on protein sequence searches against pfam and pVOG (Supplementary Data 5). The black dot denotes a pseudogene (early stop codon); domain of unknown function (DUF).

We then scanned the genomic contexts (~25 kb upstream and downstream) surrounding the *acr* homologs to identify marker genes that could provide situational insights (Supplementary Data 4). Our analyses revealed the association of the identified *acrs* with distinct types of MGEs, including phages and conjugative elements (Fig. 2d). The position and composition of *acr* loci relative to neighboring gene cassettes, were highly variable between MGEs (Supplementary Fig. 4). We observed related phage genomes harboring completely distinct *acr* loci, in some cases carrying a different *aca* gene (Fig. 3 and Supplementary Fig. 4), and sometimes lacking a known *aca* (Supplementary Fig. 5). For example, we found a number of sequenced *Pectobacterium* genomes with integrated prophages that have similarities with *Pectobacterium* phage ZF40 (a phage carrying the *acrIF8-aca2* locus)^18,20^, but in the remaining cases the prophage regions encoded different combinations of *acr*(s), together with *aca5* (Fig. 3). This observation, together with the common detection of closely related *aca* and *acr* homologs within taxonomically mixed bacterial clades (Fig. 1a and Supplementary Fig. 3, Supplementary Data 4), indicates that these genes are prone to frequent recombination events and horizontal transfer between diverse MGEs.

### Inhibition by AcrIF18* and AcrIF15 manifests upstream of target DNA binding

**Fig. 4:**
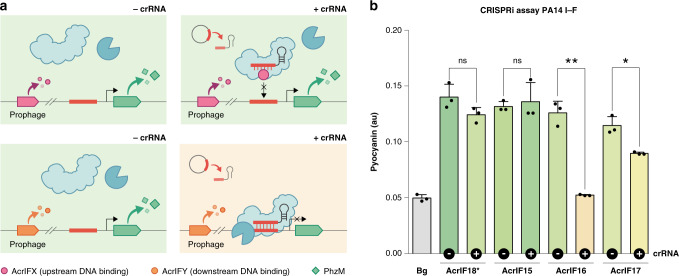
AcrIF18* and AcrIF15 prevent type I–F CRISPRi. **a** Schematic of the CRISPRi assay. In the absence of a crRNA, *phzM* is transcribed (green rhomb) and pyocyanin is produced at normal levels (green medium). In the presence of a crRNA and an Acr acting upstream of the target DNA-binding stage (purple circle), transcription of *phzM* by Cascade is de-repressed (no color change; green). If the Acr’s activity manifests downstream of the target DNA-binding stage (orange circle), *phzM* expression is repressed (color change; yellow). **b** Average pyocyanin production levels for four CRISPRi lysogens expressing a prophage-encoded Acr (AcrIF15–18*) in the presence and absence of a crRNA (indicated by the ± sign in the black circles on the *x*-axis). “Bg” represents the background pyocyanin detection levels for the assay. Error bars indicate the standard deviation of the mean for three biological replicates (unpaired two-sided *t*-test; *p < 0.05, **p < 0.005, ns = not significant; p_AcrIF18*_ = 0.1, p_AcrIF15_ = 0.7, p_AcrIF16_ = 0.0003, p_AcrIF17_ = 0.006). Representative colors displayed in the graph have been derived from pictures of the results, see Supplementary Fig. 7. Source data are available in the Source data file.

Previous mechanistic characterizations of Acr proteins have revealed a diverse range of inhibitory activities, such as interference with crRNA loading, inhibition of target DNA recognition, and inhibition of DNA cleavage^8,17^. The newly discovered Acr families present substantial differences in their biochemical properties: molecular weights (MW) ranging from 7.5 to ~20 kDa and predicted average isoelectric points (pI) spanning from acidic net charges (~pH = 4) to basic (~pH = 10) (Fig. 4d and Supplementary Fig. 6). These data, together with the observed lack of sequence homology between them, suggest potential differences in their mechanisms of action.

Using a previously established CRISPRi system^33,34^, we sought to investigate whether any of the identified inhibitors of the *P. aeruginosa* I–F CRISPR–Cas system (AcrIF15–18*) act upstream of Cascade-target DNA binding. In this CRISPRi assay, the PA14 I–F Cascade complex is crRNA-guided to represses the promoter of *phzM (phzM-*crRNA). Because PhzM is required for the production of the blue-green pigment pyocyanin, target DNA binding leads to a color change in the culture medium, from green to yellow. We constructed CRISPRi lysogens carrying DMS3m prophages expressing each Acr (AcrIF15–18*) and looked for CRISPRi inhibition (Fig. 4a). Cultures expressing AcrIF18* or AcrIF15 exhibited no significant change in pyocyanin accumulation compared to the absence of *phzM-*crRNA control (always green) (Fig. 4b, Supplementary Fig. 7). This indicates that their inhibitory activities manifest at a stage prior to target DNA binding. On the other hand, AcrIF16–17 did not block CRISPRi (a significant decrease in pyocyanin production is observed; yellow cultures) suggesting inhibition manifests downstream of DNA-binding. 

### Identification of a previously undescribed Aca and two type I–F Acrs

**Fig. 5:**
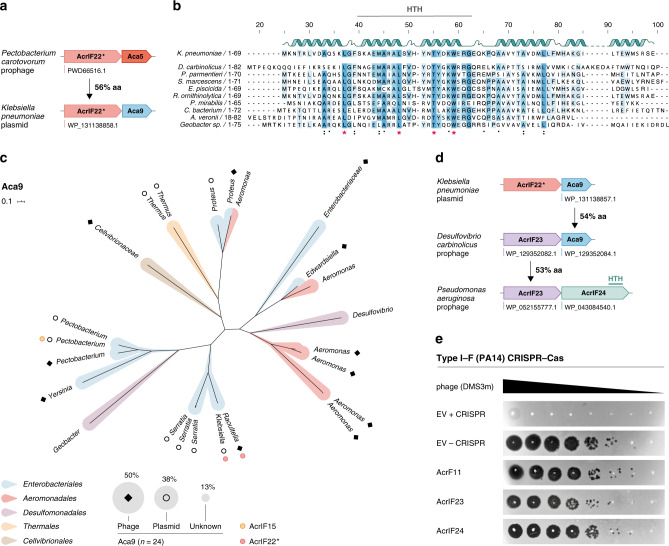
Discovery of Aca9 uncovers two potent I–F Acrs. **a** Identification of an AcrIF22* homolog reveals a previously undescribed *aca* marker gene (*aca9*). **b** Alignment of diverse Aca9 representatives carried by Proteobacterial MGEs. Predicted secondary structure alpha-helix regions are illustrated as ribbons for the *K. pneumoniae* homolog (top). The HTH DNA-binding domain is highlighted in gray. **c** Phylogenetic diversity of Aca9 across publicly available sequences. Tree branches are colored by the bacterial host from where they originate (Order level). The MGE origin is shown with an empty circle (plasmid-like elements), a black diamond (phage), and left blank when the genomic context was unclear. The relative percentage distribution of *aca9* orthologs by MGE origin is depicted as the area of the gray shaded circles in the legend. The positions in the tree where *acrIF15* and acrIF22* are found associated with *aca9* are marked with yellow and red circles, respectively. **d** Guilt-by-association searches following *aca9* led to the discovery of two candidate *acr* genes in *P. aeruginosa*. **e** Functional testing for inhibition of the PA14 I–F CRISPR–Cas system was performed by phage plaque assays in strains carrying plasmids expressing the indicated *acr* candidates. Ten-fold serial dilutions of CRISPR-targeted DMS3m phage were titered on lawns of *P. aeruginosa* naturally expressing its I–F CRISPR–Cas system. The ΔCRISPR strain shows phage replication in the absence of CRISPR–Cas targeting. AcrIF11 was employed as a positive control for strong I–F inhibition. Source data are available in the Source data file.

While exploring the genomic contexts of the identified *acrs*, we noticed that two AcrIF22* orthologs (encoded by a *Raoultella* phage and a *Klebsiella* plasmid, Supplementary Data 4) were located upstream of the same small gene of unknown function distinct from, but in the same position as, *aca5* (Fig. 5a). Motivated by this finding, we sought to investigate the potential Aca role of this gene further. A hallmark of all other previously identified Aca proteins is the presence of a helix-turn-helix (HTH) DNA-binding domain, necessary for the transcriptional repression of the *acr* operon^19,20^. Multiple sequence alignments of diverse representatives followed by functional domain prediction analyses revealed a conserved HTH motif in the hypothetical protein (Fig. 5b and Supplementary Figs. 8 and 9). Phylogenetic analyses showed a wide distribution of homologs across diverse Proteobacterial MGEs, including phages (50%) and plasmids (38%) (Fig. 5c and Supplementary Data 6). Moreover, our search revealed that this gene is also encoded downstream of an *acrIF15* homolog in a *Pectobacterium* plasmid (Fig. 5c). Taken together, these results indicate this is an anti-CRISPR associated (*aca)* gene, hereafter referred to as *aca9*.

To test whether *aca9* could be exploited as a marker for *acr* discovery, we searched for *aca9*-associated genes with homologs in *P. aeruginosa*, one of our model CRISPR–Cas organisms (Fig. 5d). We identified a *P. aeruginosa* homolog of a hypothetical protein encoded next to *aca9* in *Desulfovibrio carbinolicus* that completely inactivated the PA14 type I–F CRISPR–Cas system (AcrIF23), as did its neighbor in a *P. aeruginosa* prophage (AcrIF24) (Fig. 5e). These two Acr proteins are homologous to a number of uncharacterized proteins that are distributed across Proteobacterial classes (Supplementary Fig. 3 and Supplementary Data 7). Although no known *aca* genes form part of the *P. aeruginosa acrIF23–24* locus, the C-terminus of AcrIF24 contains an HTH domain, implying a possible dual Acr-Aca function (Fig. 5d and Supplementary Fig. 9). A similar binary role was previously shown for AcrIIA1^35^ and AcrIIA13–15^27^, suggesting that self-regulation by Acrs may be widespread. Our results reveal two additional I–F inhibitors associated with genes encoding proteins containing different HTH domains.

### Acrs cluster with Anti-RM and other anti-defense genes

**Fig. 6:**
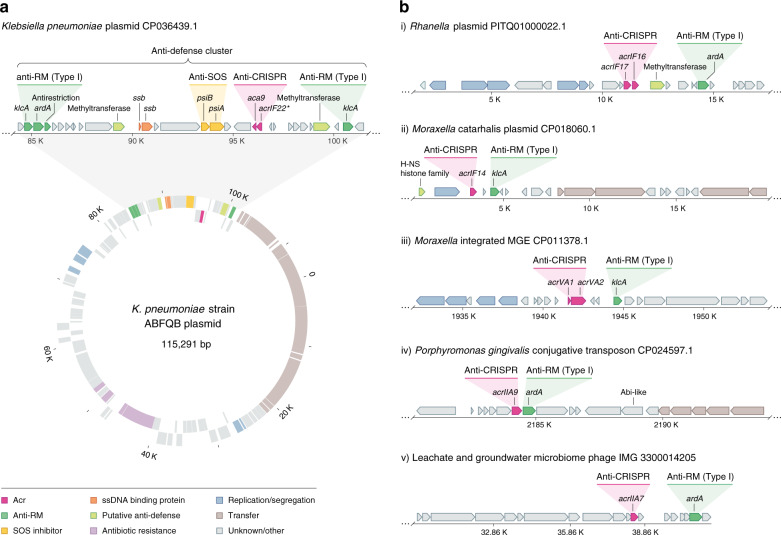
Acrs cluster with other antagonists of host defense functions. **a** Genome organization of the *Klebsiella pneumoniae* ABFQB plasmid unitig_1 (CP036439.1), highlighting the anti-defense cluster region. Genes are colored according to their predicted functions as shown in the key. Coding regions encoded on the plus and minus strands are shown on the outer and inner lanes of the gene map, respectively. **b** Examples of genomic regions within diverse MGEs where *acrs* were found in the vicinity of known anti-RM genes and other putative anti-defense determinants. Genes are colored according to the gene key in (**a**).

A closer examination of the genomic environments surrounding the newly identified *acr* loci revealed several intriguing instances of additional anti-defense system components (Fig. 6). For example, an anti-defense gene cluster was identified in a discrete region of a *Klebsiella pneumoniae* plasmid, separated from the gene modules responsible for plasmid housekeeping functions (e.g., replication, partitioning, and conjugative transfer) (Fig. 6a). Together with an aca9-acrIF22* Acr locus, we found genes encoding anti-restriction-modification systems (Anti-RM) (e.g. ArdA and KlcA^36,37^) and a plasmid SOS-inhibition (*psi*) locus involved in suppressing the deleterious host SOS response elicited by conjugative plasmid entry^38^. Orphan methyltransferase genes were also co-encoded in this region, suggesting a potential protective role against host restriction enzymes, as shown previously for other MGEs^39,40^. Furthermore, *acrIF16* and *acrIF17* were found adjacent to a methyltransferase gene and in close proximity to an Anti-RM gene (*ardA*) in a *Rahnella* plasmid (Fig. 6b(i)).

We then explored whether anti-RM genes are present nearby previously validated *acr* genes. Our analyses revealed the colocalization of the anti-RM gene *klcA* with previously described type I and V *acrs* (e.g., *acrIF14* and *acrVA1-2)* in *Moraxella* MGEs (Fig. 6b(ii), b(iii)). Moreover, a gene encoding an H-NS histone family homolog was adjacent to *acr* and anti-RM loci in a *Moraxella catarrhalis* plasmid (Fig. 6b(iii)). MGE-encoded H-NS-like proteins are often considered “stealth proteins” that tamper with host gene expression^41,42^. Given the H-NS-mediated silencing of CRISPR–Cas adaptive immunity in *E. coli*^43,44^, the lateral acquisition of H-NS homologs may help MGEs evade adaptive immunity, as previously proposed for certain plasmids^45,46^ and phages^47^. Finally, we also found close ties between type II *acrs* (e.g., AcrIIA9 and AcrIIA7) and anti-RM components in other types of MGEs, including a conjugative transposon and a putative phage (Fig. 6b(iv), b(v)).

Collectively, these results suggest that, apart from accumulating *acrs* against CRISPR–Cas systems (Figs. 1–5), MGEs compile a broader arsenal of inhibitors to overcome other host immune mechanisms. Intriguingly, the resulting collection of inhibitors appears to mirror the clustered arrangement of defense systems in their hosts, termed “defense islands”^48–50^. We found that the gene neighborhoods of loci encoding Acr and Anti-RM proteins are typically crowded with other small hypothetical protein-coding genes of unknown function (Fig. 6). We speculate that these anti-defense gene clusters may constitute an unrecognized phenomenon in diverse MGEs, potentially enriched with new genes that antagonize diverse defense systems^51^.

## Discussion

Following an *aca*-based guilt-by-association search, 11 Acr families and a new Aca family were discovered across chromosomal and extrachromosomal MGEs of mostly Enterobacteriaceae (Figs. 1b, 5d). These findings ascribe function to a dozen gene families that were previously only hypothetical and reveals that the diverse MGEs that carry them are likely encountering and evading functional CRISPR immunity in situ. The Acr proteins identified share no sequence similarity with known Acrs, increasing the collection of distinct subtype I–F Acrs to 24 (from 14) and I–E CRISPR–Cas inhibitors to 10 (from 7). Many genomes analyzed showed instances of self-targeting, where integrated MGEs carrying the *acr* operons had regions with perfect identity to host-derived CRISPR spacers (Fig. 1b, c). One self-targeting spacer was even predicted to target an *acr* gene *(acrIF16*), encoded by a *Pectobacterium parmentieri* prophage (Fig. 1c, bottom and Supplementary Data 2). We only sampled a fraction of genes associated with *aca5* and *aca9* (Figs. 1a, 5c), suggesting that many more *acrs* linked to these marker genes await discovery.

Loci encoding Acrs frequently contain more than one *acr* gene^9,12,13,23^. Here we observed loci where as many as four distinct *acrs* are “stacked” upstream of *aca5* (Fig. 1). It is unclear what fitness benefits are associated with such locus organizations and whether functional redundancy or cooperation between the different Acrs occurs. Given the fast MGE-host co-evolutionary arms race, carrying multiple *acrs* likely serves as a safeguard against Cas mutational escape or subtype diversity. Alternatively, multiple Acr proteins could provide MGEs with “division of labor” potential where different Acr proteins are used during distinct stages of the MGE life cycle, or contribute to a more robust inhibitory effect by blocking the immune pathway at different stages^52^.

Former mechanistic characterizations of Acr proteins have revealed a remarkable diversity of inhibitory functions^17^. Interestingly, relatively low MWs and pIs have been reported for Acrs inhibiting CRISPR–Cas systems via DNA mimicry^53,54^, suggesting a putative link between low MW/pI values and mechanistic inhibitory function. Given the small size and negative charges of AcrIF18* and AcrIF15 (Supplementary Fig. 6), and their CRISPRi inhibitory activities (Fig. 4b), it is possible that they function as DNA mimics—analogous to AcrIF2^55^, AcrIF10^56^, AcrIIA2^53,54^ and AcrIIA4^57^. DNA-recognition domains in Cas proteins (and components of other bacterial defense systems) are functionally constrained, making them desirable targets for broad spectrum inhibition. A great example of how MGEs exploit this “Achilles heel” is phage protein Ocr, a DNA mimic that provides protection from both type I RM systems and BREX (Bacteriophage exclusion systems)^58^. While DNA mimicry could help explain the broad inhibitory activity of AcrIF18*, further experiments are required to ascertain such functionality.

Although the Acr families we describe here are predominantly detected on prophage elements (Figs. 2d, 3, 5c), many are also carried by other types of MGEs, such as conjugative plasmids and ICEs (Figs. 2d, 5c). Our results support the notion that Acrs play an important role in facilitating the horizontal transfer of diverse MGE-encoded traits, such as plasmid-encoded antibiotic resistance determinants (Fig. 6a and Supplementary Data 8)^16^. Consistent with this idea, we recently reported a positive association of *acr* genes and acquired antibiotic resistance genes in *P. aeruginosa* genomes^59^. Interestingly, the taxonomically broadly distributed AcrIF17 is particularly enriched on conjugative plasmids (Fig. 2d). Because conjugative elements often exhibit broader transfer host ranges than phages^60,61^ these results may reveal a relationship between the MGE origin of *acrs*, their phylogenetic distribution and inhibitory spectrum.

Consistent with previous work, we show that Acrs tend to inhibit the specific CRISPR–Cas system(s) present in the hosts of the MGEs carrying them, although the inhibition spectrum of certain Acrs is occasionally broader^12,18,62,63^. These data indicate the challenge of inferring inhibitory activity a priori and highlights the necessity to interrogate *acr* function experimentally in a case by case manner using a panel of CRISPR–Cas systems.

Because the dynamics of gene flow within microbial communities are governed by the interactions between MGEs and their hosts, shedding light on the defense/anti-defense arms race is integral for understanding the ecology and evolution of bacteria. However, prokaryotes possess an extraordinary variety of defense mechanisms and identifying uncharted immune systems has proved challenging. Previous work has shown that defense systems often co-localize within defense islands in bacterial genomes^49^, thus allowing the identification of undiscovered immune systems^5,50,64^. Here, we find that *acr* loci often cluster with antagonists of other host defense functions (e.g., anti-RM) and hypothesize that MGEs organize their counter defense strategies in “anti-defense islands” (Fig. 6). In support of these observations, a previously undescribed immune evasion strategy (a double-strand DNA break repair system) and two new anti-RM genes were found located immediately adjacent to each other across several conjugative MGEs^65^. We anticipate that this genetic co-occurrence will be useful for the discovery of novel anti-defense systems.

## Methods

### Bioinformatic searches and phylogenetic analyses of Aca and Acr proteins

Protein sequences of Aca5 homologs were identified through 4 iterations of PSI-BLAST with default search parameters against the non-redundant protein database (NCBI-NR) using the *Pectobacterium* Aca5 WP_039494319.1 sequence as a query (Supplementary Data 9). Only hits with >70% coverage and e-value <10^−8^ were included in the generation of the position-specific scoring matrix (PSSM). An HMM model of Aca5 was also built using PSI-BLAST hits (three iterations; query cover: >90% and identity >55%). Acr candidates were identified following a previously described guilt-by-association approach^18^. Briefly, hypothetical ORFs upstream genes encoding Aca5 homologs were found through a combination of bioinformatic searches using PSI-BLAST (up to three iterations, only considering hits with e-values <10^−4^ for PSSM generation) and hmmsearch (HMMER v3.0; e-value cutoff 0.05; the script was modified to extract upstream and downstream nucleotide sequences for each hit).

Multiple alignments of the identified Aca and Acr proteins were performed with the MUSCLE software^66^. Alignment sites with gaps were trimmed from the alignment. Maximum Likelihood phylogenetic trees were constructed using MEGA^67^ (500 bootstraps and standard settings) and displayed using iTOL^68^.

### MGE/prophage context, CRISPR–Cas and self-targeting analyses

The genomic contexts (<25 Kb upstream and downstream) of all identified *acr* homologs were scanned manually in search for prophage, plasmid, and ICE signature protein-coding genes. The MGE type and corresponding signature gene(s) used to determine the *acr* origin are displayed in Supplementary Data 4. In the absence of annotated genes, PSI-BLAST searches (70% coverage, <10^−4^ e-value) were performed with genes neighboring the *acrs* in an attempt to find homology to genes of known function that could provide situational insights. CRISPRCasFinder^69^ and CRISPRCasTyper (https://typer.crispr.dk/)^70^ were employed to determine the presence and sequence integrity of the CRISPR–Cas systems in the genomes of the bacterial hosts encoding the selected *acr* candidates and to extract the corresponding CRISPR spacers (Supplementary Data 1). Self-targeting analyses were performed using CRISPRTarget^71^, STSS^15^, and CRISPRminer(v2)^72^ by blasting the spacer contents against the host genome assemblies (Supplementary Data 2). Prophage regions were identified and annotated via PHASTER^73^ and the position of the *acr* loci and self-targets were compared to the predicted prophage regions (Supplementary Data 2).

### CRISPR–Cas sequence identity comparisons

The protein sequences of the Cas orthologs of the model CRISPR–Cas systems tested and the endogenous CRISPR–Cas systems (hosts from which the selected *acrs* originate) were aligned using BLASTp. An average score of the percentage sequence identity between systems was calculated (Supplementary Data 3). Only the proteins involved in the interference complex were taken into consideration in this analysis (i.e., excluding the adaptation module: Cas1 and Cas2 in I–E systems and Cas1 in I–F systems).

### Bacterial strains, phages, and growth conditions

All strains and phages used in this study are listed in Supplementary Tables 1 and 2. *Pseudomonas aeruginosa* strains (PA14, PAO1) and *Escherichia coli* strains (Mach-1) were routinely grown at 37 °C in Lysogeny Broth (LB) (10 g L^−1^ tryptone, 5 g L^−1^ yeast extract, and 10 g L^−1^ NaCl). *Pectobacterium atrosepticum* SCRI1043 and *Serratia* sp. ATCC39006 were grown at 25 °C and 30 °C, respectively in LB (10 g L^−1^ tryptone, 5 g L^−1^ yeast extract, and 5 g L^−1^ NaCl). All solid plate media were supplemented with 1.5% w/v agar. Media were supplemented with antibiotics to maintain the pHERD30T plasmid (and derivatives): 30 µg mL^−1^ for *Pectobacterium atrosepticum* SCRI1043 and *Serratia* sp. ATCC39006, 15 µg mL^−1^ gentamicin for *E. coli*, and 50 µg mL^−1^ gentamicin for *P. aeruginosa*. When appropriate, the following inducer concentrations were used: 0.1–0.3% w/v arabinose and 0.1 mM isopropyl β-D-1-thiogalactopyranoside (IPTG). During heat shock transformations, *E. coli* was recovered in SOC media (20 g tryptone, 5 g yeast extract, 10 mM NaCl, 2.5 mM KCl, 10 mM MgCl_2_, 10 mM, MgSO_4_, and 20 mM glucose in 1 L dH_2_O).

*Pseudomonas* phages DMS3, DMS3m, and JBD30 derivatives were propagated on PA14 ΔCRISPR or PAO1 WT. *Pectobacterium* phage ϕTE^74^ and *Serratia* phage JS26^75^ were propagated on *P. atrosepticum* SCRI1043 (WT)^76^ and *Serratia* sp. ATCC39006 LacA strains^77,78^, respectively. *Pseudomonas* phages were stored at 4 °C in SM buffer (100 mM NaCl, 8 mM Mg_2_SO_4_, 50 mM Tris-HCl, pH 7.5, 0.01% w/v gelatin) over chloroform. *Pectobacterium* and *Serratia* phages were stored at 4 °C in phage buffer (10 mM Tris-HCl pH 7.4, 10 mM MgSO_4_ and 0.01% w/v gelatin) over chloroform.

### Cloning of candidate anti-CRISPR genes

Candidate *acr* genes identified in the *aca5*-based guilt-by-association search (Supplementary Data 1) were synthesized as gene fragments (Twist Biosciences) and cloned into the NcoI and HindIII sites of the pHERD30T shuttle vector using Gibson Assembly (New England Biolabs). The plasmid constructs were propagated in commercial *E. coli* Mach-1 competent cells (Invitrogen, Thermo Fisher Scientific) upon transformation, following the manufacturer’s recommendations. AcrIE8.1, AcrIF17.2, and AcrIF18.2* were synthesized as gBlocks (IDT) and ligated into the NcoI and HindIII sites of the pHERD30T shuttle vector and transformed into *E. coli* DH5α competent cells. The integrity of the cloned fragments were verified via Sanger sequencing using primers outside of the multiple cloning site (Supplementary Table 3). A list of the plasmids and oligonucleotides used in this study can be found in Supplementary Table 3 and 4.

### Preparation of *P. aeruginosa*, *P. atrosepticum*, and *Serratia* sp. ATCC39006 strains for *acr* functional testing

The pHERD30T plasmids with different candidate anti-CRISPRs were electroporated into the different *P. a*eruginosa strains. Briefly, overnight cultures were washed twice in 300 mM sucrose and concentrated ten-fold. Competent cells were then transformed with 50–100 ng plasmid and incubated without plasmid selection in LB broth for 1 h at 37 °C before they were grown overnight at 37 °C on LB agar plates with plasmid selection. For the transformation of these plasmids in *P. atrosepticum* and *Serratia* ATCC39006, they were first transformed into the chemical competent *E. coli* ST18 and cells were plated onto LB agar plates with 50 µg mL^−1^ 5-aminolevulinic acid (ALA). Then, the donor ST18 cells were conjugated with recipient *P. atrosepticum* (PCF188)^18^ and *Serratia* ATCC39006 PCF524 (I–E) and PCF525 (I–F). The positive clones were selected by streaking onto LB agar plates containing 30 µg mL^−1^ gentamicin. The arabinose-inducible promoter in pHERD30T was used to drive the expression of the candidate *acr* genes.

### *P. aeruginosa* phage immunity assays

The functionality of the identified *acr* candidate genes was assessed through phage spotting assays or efficiency of plaquing (EOP). These tests evaluated the replication of CRISPR-targeted phages DMS3m (I–F) and JBD30 (I–C and I–E) in bacterial lawns relative to the empty vector control. Efficiency of plaquing (EOP) was calculated by dividing the number of plaque-forming units (pfus) formed on a phage-targeting strain by the number of pfus formed on a non-targeting strain: ΔCRISPR strain (I–F and I–E) or the absence of CRISPR expression inducer (I–C). Additional controls included infection in the presence of an already validated Acr (i.e., AcrIE4/F7, AcrIF11, or AcrIC1). Each pfu calculation was performed in three biological replicates and expressed as the mean EOP ± SD (error bars). Briefly, 200 µL of bacterial overnight cultures were mixed with 10 µL of ten-fold phage serial dilutions and combined with 4 mL of molten top agar (0.7%) supplemented with 10 mM MgSO_4_. The mix was poured onto LB agar (1.5%) plates containing 50 µg mL^−1^ gentamicin and 10 mM MgSO_4_. Additionally, plates were supplemented with 1 mM IPTG and 0.3% w/v arabinose for experiments using the PAO1 strain (I–C CRISPR–Cas) and with 0.3% w/v arabinose when using the PA4386 strain (I–E CRISPR–Cas), and PA14 strain (I–F CRISPR–Cas). Plates were incubated overnight at 30 °C and phage plaque-forming units (pfu) were calculated. In phage spotting experiments, phage dilutions 3.5 µL of ten-fold serial dilutions of the phage lysates were spotted onto the plate surface containing the bacterial lawn in the top agar. Plate images were obtained using Gel Doc EZ Gel Documentation System (BioRad) and Image Lab (BioRad) software.

### Anti-CRISPR Assay in *P. atrosepticum* and *Serratia* sp. ATCC39006

PCF188, PCF524, and PCF525 were transformed with plasmids expressing the different Acrs and overnight cultures were used to pour top agar plates (100 µL culture added to 4 mL LB with 0.35% agar) onto LB agar plates (containing 30 µg mL^−1^ gentamicin and 0.1% w/v arabinose). Due to the toxic effects on *Serratia* cells, AcrIF9 (positive control for inhibition) was tested in the absence of arabinose. Twelve-fold serial dilutions of phage ϕTE and JS26 lysates were made in phage buffer, and 15 µL was spotted on the dried top agar plates. Then, the plates were incubated overnight at 25 °C for *Pectobacterium* and 30 °C for *Serratia*. The efficiency of plaquing (EOP) was determined by calculating the pfus per mL of the *Pectobacterium* 3 × TE and *Serratia* cells (expressing the different Acrs) divided by the pfus of the corresponding wild-type with an empty vector pHERD30T control. Three biological replicates were done for each of the experiments (Source data file).

### Construction of recombinant *acr* phages

DMS3m phage derivatives encoding AcrIF15, AcrIF18*, AcrIF16, and AcrIF17 were constructed. Briefly, a recombination plasmid with homology to the DMS3m acr locus^78^ was used to clone the Acr genes of interest upstream of *aca1* by Gibson Assembly (New England Biolabs). The resulting vectors were used to transform PA14 ΔCRISPR and the strains were infected with WT DMS3m::gent35 cassette. Phages were recovered after full plate infections in selection plates containing 50 µg m^−1^ L gentamicin and the resultant phage lysates were used for full plate infections in plates without selection. Recombinant phages were sequentially passaged through PA14 ΔCRISPR 3 times to purge away potential non-recombinant phage carryover. The integrity of the cloned Acr genes were verified by Sanger sequencing. Phages were stored in SM buffer at 4 °C.

### Generation of recombinant Acr phage PA14 (dCas3) lysogens

200 µL of PA14dCas3 overnight culture was added to 4 mL of 0.7% LB top agar and spread on 1.5% LB agar plates supplemented with 10 mM MgSO_4_. 3 µL of the recombinant *acr* phages were spotted on the top agar bacterial lawns and plates were incubated at 30 °C overnight. Following incubation, bacterial survivors (lysogens which undergo superinfection exclusion or are surface receptor mutants) within the plaques were isolated and spread on 1.5% LB agar plates. Single colonies were assayed for phage resistance by streaking across a line of phage lysate, compared to a sensitive WT PA14 control. Prophages were confirmed by sequencing, checking for resistance to superinfection by the same phage, and assessing the spontaneous production of phage from the lysogenic strain (supernatant was titered on PA14 ΔCRISPR).

### CRISPRi-based pyocyanin repression assay in *P. aeruginosa*

The pyocyanin repression assay was carried out using PA14dCas3 lysogens harboring the constructed DMS3m prophages expressing either AcrIF15, AcrIF16, AcrIF16 or AcrIF18*. The different were transformed with pHERD30T::crRNA*phzM*, a vector expressing a crRNA designed to target the promoter of *phzM*, a chromosomal gene in PA14 which is involved in the production of the pyocyanin (blue-green pigment)^34^. In the presence of crRNA *phzM* CRISPRi repression leads to a color change in the culture medium, from green to yellow. The different lysogens were additionally transformed with an empty vector (pHERD30T), serving as a non-targeting control (remains green).

Cultures of three independent lysogens were grown overnight in LB supplemented with gentamicin (50 µg mL^−1^) for vector retention and 0.3% arabinose to induce crRNA expression. Pyocyanin was extracted from the overnight cultures and quantified by measuring absorbance at 520 nm^34^. Representative pictures of the color changes are also displayed (Supplementary Fig. 7).

### Software and statistical analysis

Numerical data were analyzed and plotted with GraphPad Prism 6.0 Software. HHPred searches were carried out for the prediction of protein domains (e.g., HTH). Protein secondary structure predictions were carried out by JPred4^79^ and Phyre2^80^. Detection of antibiotic resistance genes was performed via BLAST against the Comprehensive Antibiotic Resistance Database (CARD)^81^ (Supplementary Data 8). A list of the Software is provided in Supplementary Table 5.

### Statistics and reproducibility

Statistical parameters are specified in the figure legends. All experiments were repeated at least two times independently with similar results.

### Reporting summary

Further information on research design is available in the Nature Research Reporting Summary linked to this article.

## Supplementary information

Supplementary Information

Description of Additional Supplementary Files

Supplementary Data 1

Supplementary Data 2

Supplementary Data 3

Supplementary Data 4

Supplementary Data 5

Supplementary Data 6

Supplementary Data 7

Supplementary Data 8

Supplementary Data 9

Reporting Summary

## Data Availability

All data used for generating the Figures and the Supplementary Figures presented in this study are available in this article. Links to public datasets used: Comprehensive Antibiotic Resistance Database (CARD); NCBI-NR; IMG/VR. Source data are provided with this paper.

## Source data

Source Data
