# Supplementary material for: Discovery of multiple anti-CRISPRs highlights anti-defense gene clustering in mobile genetic elements
Source: Nat Commun. 2020 Nov 6;11:5652. doi: 10.1038/s41467-020-19415-3 (PMC7648647; doi:10.1038/s41467-020-19415-3)
Supplement: Supplementary file 1 — Supplementary Information [file 41467_2020_19415_MOESM1_ESM.pdf]

# Supplementary Information

## Discovery of multiple anti-CRISPRs uncovers anti-defense gene clustering in mobile genetic elements

Pinilla-Redondo *et al., 2020*

## Supplementary Tables

### Supplementary Table 1. Bacterial strains used in this study

| Name | Source | Identifier/Reference |
|---|---|---|
| *Escherichia coli* DH5α-cloning strain | Fineran Laboratory | Gibco/BRL |
| *Escherichia coli* Mach-1 | Thermo Fisher Scientific | Cat #C862003 |
| *Escherichia coli* ST18-Auxotrophic donor for biparental conjugation | Fineran Laboratory | Ref. (1) |
| *Pectbacterium atrosepticum* (PCF188) with 3 spacers to target ϕTE (I-F) | Fineran Laboratory | Ref. (2) |
| *Pectobacterium atrosepticum* SCRI1043 | Fineran Laboratory | BX950851.1 |
| *Pseudomonas aeruginosa* dCas3 PA14 | Laboratory of George O'Toole | Ref. (3) |
| *Pseudomonas aeruginosa* PAO1 | Laboratory of Alan Davidson | Refseq: NC_002516.2 |

| | | |
|---|---|---|
| *Pseudomonas aeruginosa* PAO1 (I-C CRISPR-Cas); LL77 | Bondy-Denomy Lab | Ref. (4) |
| *Pseudomonas aeruginosa* SMC4386 | Laboratory of George O'Toole | LOQZ00000000 |
| *Pseudomonas aeruginosa* UCBPP-PA14 WT | Laboratory of Alan Davidson | Refseq: NC_008463.1 |
| *Pseudomonas aeruginosa* UCBPP-PA14 ΔCRISPR1ΔCRISPR2 (SMC5454) | Laboratory of George O'Toole | Ref. (3) |
| *Serratia* sp. ATCC39006 | Fineran Laboratory | NZ_CP025084.1 |
| *Serratia* ATCC39006 (PCF524) with 1 spacer to target phage JS26 (I-E) | Fineran Laboratory | Unpublished |
| *Serratia* ATCC39006 (PCF525) with 1 spacer to target phage JS26 (I-F) | Fineran Laboratory | Unpublished |

**Supplementary Table 2. Bacteriophages used in this study**

| **Name** | **Source** | **Identifier** |
|---|---|---|
| *Pectobacterium* Phage ϕTE | Fineran Laboratory | Ref. (5) |
| *Pseudomonas* phage JBD30 | Laboratory of Alan Davidson | Refseq: NC_020298.1 |

| | | |
|---|---|---|
| *Pseudomonas* phage DMS3m (DMS3 derivative) | Laboratory of George O'Toole | Refseq (DMS3): NC_008717.1 |
| *Pseudomonas* phage<br>DMS3m$_{acrIF15}$<br>DMS3m$_{acrIF16}$<br>DMS3m$_{acrIF17}$<br>DMS3m$_{acrIF18*}$ | Bondy Denomy Laboratory | This study |
| *Serratia* Phage JS26 | Fineran Laboratory | Ref. (6) |

**Supplementary Table 3. List of oligonucleotides used in this study.**

| Name | Sequence | Description | Reference |
|---|---|---|---|
| PF138 | CACACTTTGCTATGCCATAG | Forward Primer to sequence pPF1896, pPF1897, pPF1898 | Ref. (7) |
| PF301 | GCTATTACGCCAGCTGGCGA | Reverse Primer to sequence pPF1896, pPF1897, pPF1898 | This study |
| QB0068 | ATGCCATAGCATTTTTATCC | Forward primer pBAD pHERD30T | https://www.quintarabio.com/ |
| Forward 108 | tgtttaactttaagaaggagatatacatacc | Forward primer to amplify *acr* gene fragments for Gibson Assembly | This study |
| Reverse 108 | ttcccagtcacgacgttgtaaaacgacggccagtgcca | Reverse primer to amplify *acr* gene fragments for Gibson Assembly | This study |
| AcrIE8.1 | TCGTCTTCACCTCGAGAAATCCCATGGCTATGACTACAATCACTATCAATACTTATGACCCTGAAGCACGTTTCAACATGGACAAGGACGAAGCCAAGTCTTTCTTTGAGTTCGTTGAAAAGAAAGCCACAGACGCAGGGTTTAATGTTCAGTACGACAGTTGCAACTATGTCGATGAAGAAA | gene fragment | This study |

| | | | |
|---|---|---|---|
| | GTGAGCGCTTTGTTGAAAAA<br>TGCTTTGAGGATTATTAAGCT<br>TCCTGTTGATAGATCCAGTAA<br>TGAC | | |
| AcrIE8.2 | tgtttaactttaagaaggagatatacatacc<br>ATGACCACAATCACCATCAA<br>CACTTATGATCCTGAAGCTC<br>GCTTCAATATGAGCGGAGAA<br>GAAGCAAAAGAATTCTTTGC<br>CTTTGTTGAAGAGCAGGCAA<br>AGGTATCAGGTTTTGATGTCT<br>ATTATGACAGTTGCACCTATG<br>TCGACGAAGAGAGTGAGCGC<br>TTTGTTGAAAAATGCTTTCAG<br>AACTACTAAtggcactggccgtcgtttt<br>acaacgtcgtgactgggaa | gene fragment | This study |
| AcrIF15 | tgtttaactttaagaaggagatatacatacc<br>ATGACTACCATCACCATTGC<br>GTACGAAGTATCTAACGACA<br>AAGTTGAAACCATCAAGACA<br>ATGGTGGAGAGCCAGCAGAT<br>TCATAACGTTAACTTTAACGG<br>CGAGGAGTTCACCATAGAGC<br>GCGGTGACTTTACCTCCATC<br>GATAAAGATGAGGCTGAGCA<br>TGTAAAACTTCTCAACAAAAT<br>TCAGGACATCATTCACGGGT<br>ACAGCTAAtggcactggccgtcgtttt<br>acaacgtcgtgactgggaa | gene fragment | This study |
| AcrIF16 | tgtttaactttaagaaggagatatacatacc<br>ATGTCATTATCTGACAAAAAA<br>GAACAGAAAGAGGCTTATCT<br>TGATGCGTTGCGCATCGCTC<br>CGCTTGATAGAGGCGTTCTC<br>AAACGCATTCATGCTGTCAAT<br>GACAACACGTTAGATAAATG<br>GCTTTACGTTGCTGATAGATA<br>TCCCACGTTTAGAGCATGTT<br>GGGAATTGTGGATGTTCCAG<br>CGTAAGCGCCGAGTACTCAT<br>ATCGAGAAAACTTCATGTTCT<br>AATCAACAGATCCACGAACA<br>GAACAATAGAGGCGTTCGAA<br>AAAACCTACCCGCCCGAAGA<br>GAGGGTTGTGGGAAAATCAT<br>ATCGGGATCTGGTAACGGAA<br>AAGGGAGAGCGTTCCGCCAA<br>CATGTATATCATTAACGGGG | gene fragment | This study |

| | | | |
|---|---|---|---|
| | AAGTAGTTGGCGCTAAAGAT<br>GCGAGCATTTTGCTCGGATA<br>CTCATCATACAACACTCTTTA<br>CGCAAAAATGAAACGACTCG<br>GAATACAGCCAGGCGACGAC<br>ATTTCACATCTAAAGCCGGAA<br>AAAAGAGGAAGAAAAAAAGA<br>ATGCAGCTAAtggcactggccgtcg<br>ttttacaacgtcgtgactgggaa | | |
| AcrIF17.1 | tgtttaactttaagaaggagatatacatacc<br>ATGGCTTCCGAATTAGAAAG<br>TTTTATCAAACGAGACAACGC<br>GCGATACGTTGCAAGTTTCA<br>GCAATTCAGGAAAAGTTCCA<br>AAAGAAGATTTTTATAGCCGT<br>GTACACCTGGTCGCTCAGTG<br>GTGCCAGACGGAGAGCGTT<br>GCCAGTTATGACGATCTGTA<br>CACCGCTGCTTATAACGATC<br>TCGTCGCCGAAATTAACGCG<br>TCAGGTCTGATAATCGAAAAT<br>TAAtggcactggccgtcgttttacaacgtc<br>gtgactgggaa | gene fragment | This study |
| AcrIF17.2 | TCGTCTTCACCTCGAGAAAT<br>CCCATGGCTATGACTGAACT<br>CGACCTGAGAAAACTCAGCA<br>AAAAGGCTTTGCTTCAGTTAA<br>TTTCTGAAGCCGCAAACGAG<br>CTGCACCGCCGTGAATCCTC<br>ATCTTCTTTTGTCGAACCGGC<br>CGCAGCAGAAGAGACTGCCC<br>TGTCACCCGGACAGGCCGAT<br>CTGGTTTTTATTAATAACTGC<br>CTGAACGCAAGTGATTATGT<br>GCATGCTGATGCAAAAGACC<br>GATACAAACAGTTAGCATCTA<br>AATACTCAGCGTGGTTCGCG<br>TACAAGGGATATCCGCGAGA<br>TTTGCGTGGTTCAGACCTCA<br>AAAAATGGAAACAGTATTTCA<br>GGCCCAACACGAGAGAACAG<br>TAAGCTTCCTGTTGATAGATC<br>CAGTAATGAC | gene fragment | This study |
| AcrIF18.1* | tgtttaactttaagaaggagatatacatacc<br>ATGACTACTATCAAAGCCGC<br>TTACATCAGCAAAGACCAGA<br>ACTGGAACGACGGCACCACC<br>ACTTATTGGTTTGACGTAAAT<br>GGTGAAACCTTTGGGGTTGT | gene fragment | This study |

| | | | |
|---|---|---|---|
| | GCATGGTGGTGAAAGCTGGA<br>ATGCCAAGGTAGTCGATTGC<br>GACGGAGCGCCATCCGACC<br>AATACACCGTTGACCAGTTC<br>AACATCACCGAAGATATGAT<br>CGCCGAATAAtggcactggccgtcg<br>ttttacaacgtcgtgactgggaa | | |
| AcrIF18.2* | TCGTCTTCACCTCGAGAAAT<br>CCCATGGCTATGACTACTAT<br>CAAAGCCACTTACATCAGCA<br>AAGACCAGAACTGGAACGAC<br>GGCACCACCACCTATTGGTT<br>TGATGTAAACGGTGAAACATT<br>CGGGGTTGTGCATGGCGGT<br>GAAAGCTGGAATTCCAAGGT<br>GGTTGACTGCGACGGAACGC<br>CATCAGACCAATACACCGTT<br>GACCAGTTCAACGTAACCAA<br>AGAGATGATCGCCGAATAAG<br>CTTCCTGTTGATAGATCCAGT<br>AATGAC | gene fragment | This study |
| AcrIF19 | tgtttaactttaagaaggagatatacatacc<br>ATGAAACCTCTCCACACCAT<br>GAATTACGATAACAATCAAAT<br>GTCGTTAGTCTATGAGAGCT<br>ACGACGAATACGGCTTTGAA<br>TATAGCGTTAAACTAAAAATT<br>TCAGTACGAGACTATCGGGG<br>TATTGATGTCAGTGCATTCAA<br>TGCATTTCCTGAATGGGAAG<br>ACACTCTGCGTATGAGAGAT<br>CGCGTCATGTCAGTAGAGGA<br>AATCGAAAATGCAATGATTTC<br>TAGATACAAGTCGCTTTTTAT<br>TGCGCCGCCAGATTGTACTT<br>ATGAGTTTGATATCTAAtggcac<br>tggccgtcgttttacaacgtcgtgactggga<br>a | gene fragment | This study |
| AcrIF20.1 | tgtttaactttaagaaggagatatacatacc<br>ATGACAAAATCAGAAATATTC<br>AAATTTGCATGGGTGGATGC<br>TCATTATCTGGCTACCACGCT<br>TGGCGGGAATGCCGTGGAAT<br>ACTTCGCTGAGTGCCTCAAA<br>AAATCTCACATGATAAACCGC<br>ACAACAGCGGTTTCTTTTGAA<br>CAAAAGGAGTATGCGGTTGA<br>CGTTGCTTATGCAGCTATAAC<br>AATCCTCGCTGATGGCGCGG | gene fragment | This study |

| | | | |
|---|---|---|---|
| | TACTAGCAAGAGCAGCGGGTAAAAACGGCTACGCAGATGCAGCTATGATTGCTATCGCTAAAATTAAACAAATGAAAATAGCTCGTCAGATTCTTGATTCCTTGATCGGAGTGAATTTCCGCAAGGGGAGCGACTTAAAATTATGGCTATCAAGCAACTGAtggcactggccgtcgttttacaacgtcgtgactgggaa | | |
| AcrIF20.2 | tgtttaactttaagaaggagatatacataccATGACTAAGGCAGAAATATTCAAATCAGCATGGACAGACGCCCACTACTGCGCTTCAGTAATGGGCGGGAAAGCTAAAGAATACTTCGCTGAATGTCTTAAGAAATCTCACATGTTAAACAGAATTAATGGTGCCTCCTTCAAGCAGAAGGAATATGCAGTCGATGTGGCATACAAGGCTATTTCTGTTCTTGCGGAGGGGGCATGTCAGGCAATAATCGCAAATAAGGACGCTTACGCAAGCGCTGCTTATCTCGCCATCGCAAAGATCAACAAAATGACATCTGCCAAACAAATTCTTGACTCACTGCTGAACGTTAATTTCCACAAGGGATTTCAACTGAAAGAGTGGCATTCAAAAAATTAAtggcactggccgtcgttttacaacgtcgtgactgggaa | gene fragment | This study |
| AcrIF21 | tgtttaactttaagaaggagatatacataccATGACGAGCATTAAAACCATTGAATACAAAGGCAACACGCTGACATTGCAGAAAGATAGCGACGCTGACTGCATCAGTTATGAGATTTTACTACGCGACCCAATCAAACACTGGGTAGGACGAGATGTTCAGATTTTAAATATTGATTCATCGACCAGTGTTGACGTCTTCTATAAGAAATTCAGCGACTCAGAACTTGCATCTATGATGCAGAGGGAATACGACGACTGCTTTCCTCTTCAAGTCACCGCAACATTAGAAAGCGTTATTGAAGCTGGCGACATTAACACAAAATGGTTTGTGGTAAATGATGAAACTGGTGCGTTATCTGGTAAATACGGTAGCGT | gene fragment | This study |

| | | | |
|---|---|---|---|
| | TTACACAGAAAGTGGCTGGTACATTTGTAATGAATCAGGTGAAAAAATTGAAAATAAACTCACTGAAAACCAAATCAGCTCAATCAGGGTAGCAATGGATAGAGGTGATACAGCAACCTATTCGTGGGAATTCGATGACTGAtggcactggccgtcgttttacaacgtcgtgactgggaa | | |
| AcrIF22* | tgtttaactttaagaaggagatatacataccATGAGCAAGAAATTCTTGGAAATCGTGGGTAACGCATCAACTAGCGCAACGTTTAACGGGAAACTGATTGGGCACAATGTCAATGCCTCCGCCTACGAAAAGACGGTGAAATCATTATCCACCTGGAAACAAATGGATCTCGCTGGAAATCATCGCCAGAAGTAAGGATGACAAAAGAGGAATATGATTCGTTTTGCGAAAAACAATCTCGCCCTCTATTCGTTCGCGGCATTGAGTTATTTGGCGCAGAAGCCCTGTTGAGTTAAtggcactggccgtcgttttacaacgtcgtgactgggaa | gene fragment | This study |
| AcrIF23 | tgtttaactttaagaaggagatatacataccATGACCAATTTCCAGACCTGGCTCGACAGTGCCGACATTCCCGTCCAGCAGAATGGGCAGTGGATTGACCTCGAAACCGGGATCGCCTATGACCCGTCGTACAACTACGCCGCGAACACCCGCCGGGCCTCTTTGAGCCCGCGCGGCATTGACGCTCGCGCCGTGGCAAAGACCTTCGGCGGTCGCGCCCTCACCGGCACAGCCAGGCAGAAGGAGTGGGCCGAGAAGATCCGCGCCGAGAAGGTACAGCAGATGAACCAAGACCAGGCGGAAATGGCCTGCGATCCAAGCGGCCTGCTCACTGCCGCCAAATTCTGGATCGAAAATCGCAACGATAGTGCTCAGGAAATCGCCGGATTCGTAATGCAGCAGAAAGCCTTGCTTGCCCAGCATCGCTCTGCCAAGGCCGCCGGGCAAGCCGACAAAGTGGCGAAAATCGCTGCTGAGTACAACGCGCTTACCGCTCGCTGGGGGT | gene fragment | This study |

| | | | |
|---|---|---|---|
| | TCTGAtggcactggccgtcgttttacaacgtcgtgactgggaa | | |
| AcrIF24 | tgtttaactttaagaaggagatatacatacc ATGAACGCCATCCATATCGG GCCGTTCTCGATCACTCCGG CTGCCCGCGGACTGCATTAC GGGGGCCTGCCGCATCACC AGTGGACCCTATACTACGGG CCCCGGGAAATGGCGATAAA GACCCTACCGGACAGTTACA CCTCGTCGGAGGTGAGGGA CGAGTTCTCAGACATCATCG CCGAGTTCGTCATCGACGCC CGGCACCGATACGCGCCCG ATGTTCTGGAACTGGTGAAC TCGGATGGTGACGCAGTGCT CGCGCGAGTCGCCGTGAGC CGACTGCCAGAAGCGTTGTC CGGGTGCATACCAGACGATC GATTCCCATACTGGCTCCTG ACCGCCAGCCGCCCACGGC TCGGGCTCCCTGTCACCCTG AACGAGTACACCGCGCTCGC GGTCGAACTCAGCGCCCCTC CACTTGCATGGATCACAGGG CTCCTCCCTGGCGAGGTACT GACACATGACGCCGAGGAGT GGCGACCGCCGACCAGTTG GGAGCTACGCCACGTTGTCG GCGAGGGGTCGTTTACTGGC GTAAGCGGCGCCGCTGCGG CCGCTCTGCTCGGAATGTCC GCAACGAATTTCCGAAAGTA CACAGCCGGGGACTCTGCC GCGAATCGCCAGAAAATCAG TTTCGCAGCCTGGCACTACC TACTCGACCGGCTCGGCGTG AAGCGGGCGAGCTGAtggcact ggccgtcgttttacaacgtcgtgactgggaa | gene fragment | This study |

**Supplementary Table 4. Plasmid constructs used in this study**

| Plasmid Name | Backbone | Description | Reference |
|---|---|---|---|
| pPF1896 | pHERD30T | p30T-acrIE8.1 (restriction cloning) | This study |
| pPF1897 | pHERD30T | p30T-acrIF18.2* (restriction cloning) | This study |

| | | | |
|---|---|---|---|
| pPF1898 | pHERD30T | p30T-acrIE17.2 (restriction cloning) | This study |
| pRPR22 | pHERD30T | p30T-acrIF18.1* | This study |
| pRPR2 | pHERD30T | p30T-acrIF15 | This study |
| pRPR6 | pHERD30T | p30T-acrIF16 | This study |
| pRPR7 | pHERD30T | p30T-acrIF17 | This study |
| pRPR1.2 | pHERD30T | p30T-acrIE8.2 | This study |
| pRPR8 | pHERD30T | p30T-acrIF21 | This study |
| pRPR11 | pHERD30T | p30T-acrIF19 | This study |
| pRPR12 | pHERD30T | p30T-acrIF20.1 | This study |
| pRPR13 | pHERD30T | p30T-acrIF20.2 | This study |
| pRPR16 | pHERD30T | p30T-acrIF22* | This study |
| pRPR36 | pHERD30T | p30T-acrIF23 | This study |
| pRPR50 | pHERD30T | p30T-acrIF24 | This study |
| pJZ_299 | pHERD30T | p30T-acrIF11 | Ref. (4) |
| pJZ_298 | pHERD30T | p30T-acrIE4-IF7 | Ref. (4) |
| p30TcrRNA | pHERD30T | p30T:crRNA*phzm* | Ref. (8) |
| NM102 | pHERD30T | p30T-acrIC1 | Ref. (4) |

**Supplementary Table 5. Software and algorithms**

| **Program** | **Source** | **Reference** |
|---|---|---|
| CRISPR/Cas finder v4.2.17 | https://crisprcas.i2bc.paris-saclay.fr/ | Ref. (9) |
| MegaX | https://www.megasoftware.net/ | Ref. (10) |
| CRISPRTarget (January 2020) | http://crispr.otago.ac.nz/CRISPRTarget/crispr_analysis.html | Ref. (11) |

| | | |
|---|---|---|
| Graphpad Prism 6.0 | www.graphpad.com | GraphPad Software, La Jolla California USA. |
| Adobe Illustrator v24.0.2 | https://adobe.com/products/illustrator | Adobe Inc., 2019. Adobe Illustrator |
| iTOL v5.6.3 | https://itol.embl.de/ | Ref. (12) |
| HHPred (February 2020) | https://toolkit.tuebingen.mpg.de/tools/hhpred | Ref. (13) |
| Phyre2 | http://www.sbg.bio.ic.ac.uk/~phyre2/html/page.cgi?id=index | Ref. (14) |
| HMMER3.0 | https://www.ebi.ac.uk/Tools/hmmer/ | Ref. (15) |
| Easyfig 2.2.2 | https://mjsull.github.io/Easyfig/ | Ref. (16) |
| IMG/VR (March 2020) | https://img.jgi.doe.gov/vr/ | Ref. (17) |
| CRISPRCasTyper v1.1.5 | https://typer.crispr.dk/ | Ref. (18) |
| JPred4 | http://www.compbio.dundee.ac.uk/jpred/ | Ref. (19) |

# Supplementary Figures

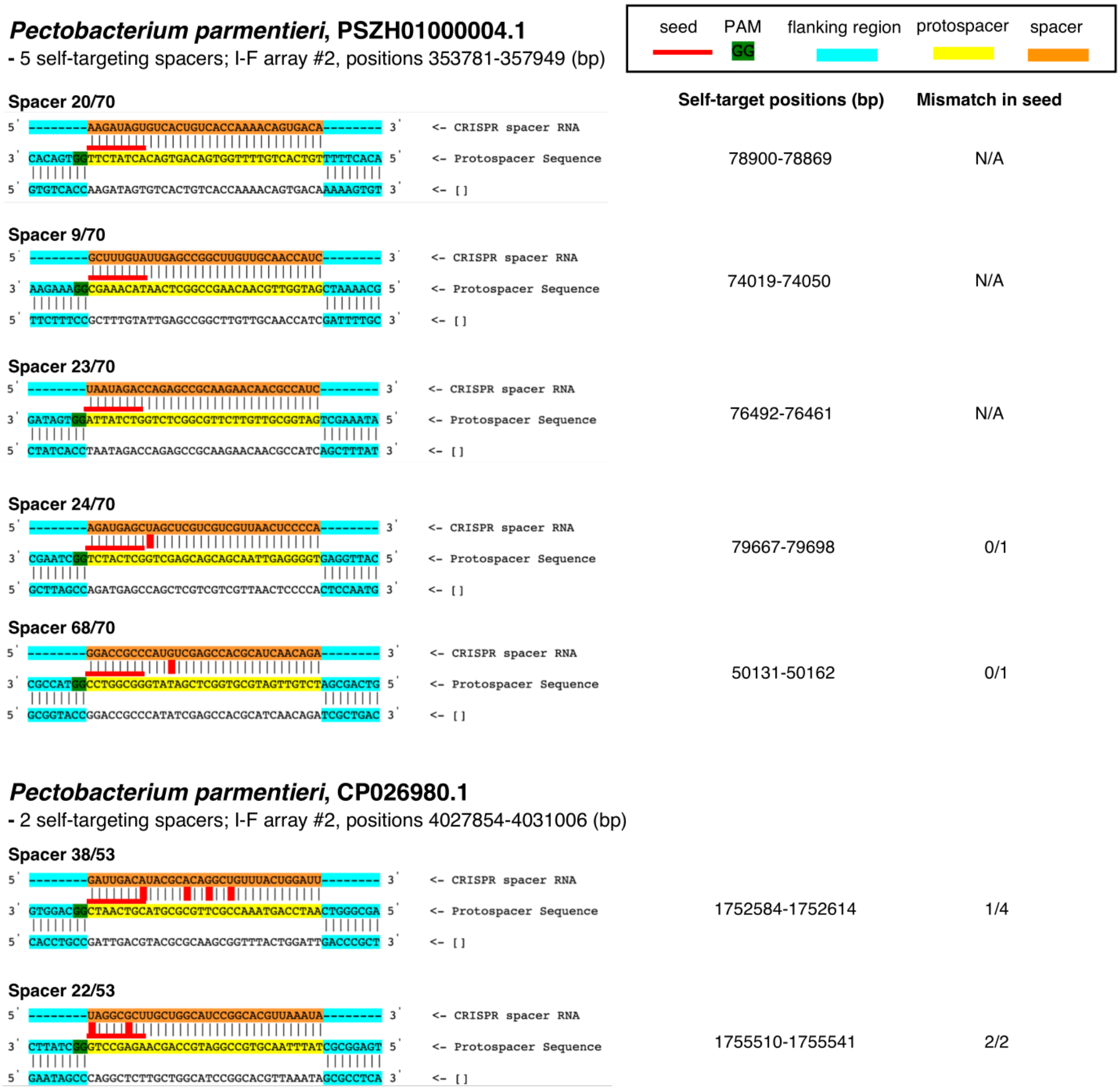


**Supplementary Fig. 1.** Spacer-protospacer match analysis for all self-targeting spacers displayed in the examples of Fig.1c. The positions of the CRISPR array and self-targets are indicated for the two *P. parmentieri* strains (sequence accession numbers indicated). The fraction of mismatches within the seed region is summarized at the right. N/A indicates that the seed region mismatch analysis is "not applicable" due to the absence of mismatches. Mismatches are shown as vertical red rectangles, protospacers in yellow, flanking regions are shown in light blue, the 8 nt long PAM-proximal seed regions are highlighted in red, I-F PAM (GG) in green and the spacer sequences in orange. Detailed information about the self-targeting spacer analyses can be found in

Supplementary Data 2. Note: spacer 22/53 has one mismatch which does not contribute to basepairing (in the 6th position) and hence only 1 functional mismatch in the seed region is considered.

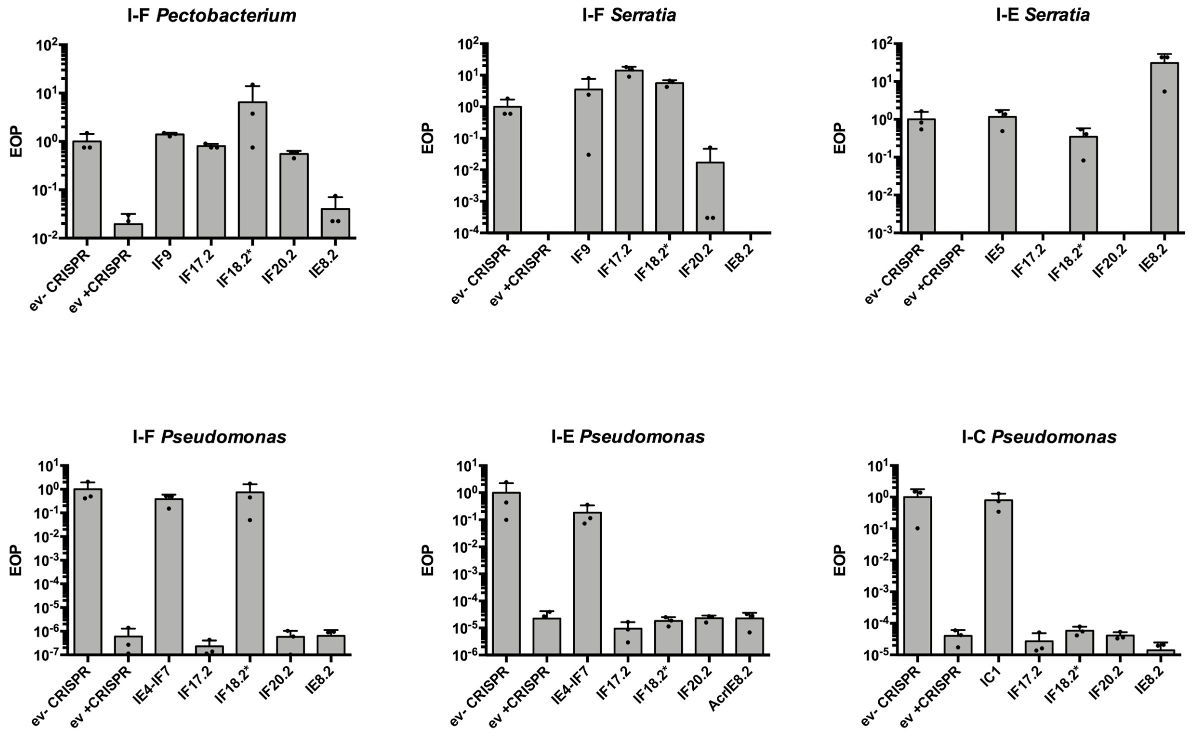


**Supplementary Fig. 2.** Functional screening of homologs of the acr candidates displayed in figure 2c. Plots show the relative efficiency of plaquing (EOP) of phages in bacterial lawns expressing the different *acr* candidates compared to EOP of the same phage in non-targeting bacterial lawns carrying the empty vector (ev). Asterisk denotes that the Arc in question is a dual I-F and I-E inhibitor. Data are presented as mean ± SEM (n = 3 biological replicates). Source data are available in the Source Data file.

## a. AcrIE8

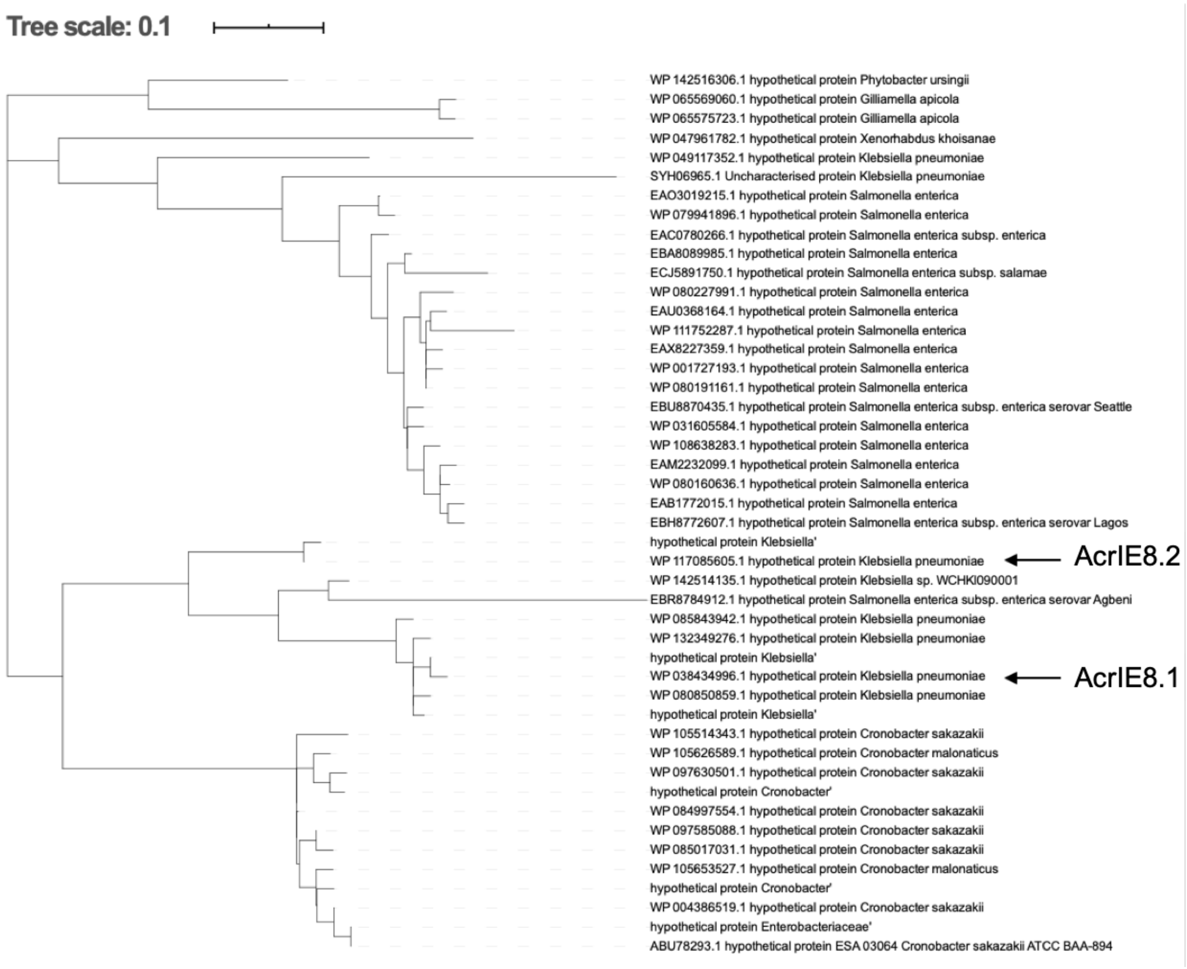
Tree scale: 0.1
WP 142516306.1 hypothetical protein Phytobacter ursingii
WP 065569060.1 hypothetical protein Gilliamella apicola
WP 065575723.1 hypothetical protein Gilliamella apicola
WP 047961782.1 hypothetical protein Xenorhabdus khoisanae
WP 049117352.1 hypothetical protein Klebsiella pneumoniae
SYH06965.1 Uncharacterised protein Klebsiella pneumoniae
EAO3019215.1 hypothetical protein Salmonella enterica
WP 079941896.1 hypothetical protein Salmonella enterica
EAC0780266.1 hypothetical protein Salmonella enterica subsp. enterica
EBA8089985.1 hypothetical protein Salmonella enterica
ECJ5891750.1 hypothetical protein Salmonella enterica subsp. salamae
WP 080227991.1 hypothetical protein Salmonella enterica
EAU0368164.1 hypothetical protein Salmonella enterica
WP 111752287.1 hypothetical protein Salmonella enterica
EAX8227359.1 hypothetical protein Salmonella enterica
WP 001727193.1 hypothetical protein Salmonella enterica
WP 080191161.1 hypothetical protein Salmonella enterica
EBU8870435.1 hypothetical protein Salmonella enterica subsp. enterica serovar Seattle
WP 031605584.1 hypothetical protein Salmonella enterica
WP 108638283.1 hypothetical protein Salmonella enterica
EAM2232099.1 hypothetical protein Salmonella enterica
WP 080160636.1 hypothetical protein Salmonella enterica
EAB1772015.1 hypothetical protein Salmonella enterica
EBH8772607.1 hypothetical protein Salmonella enterica subsp. enterica serovar Lagos
hypothetical protein Klebsiella'
WP 117085605.1 hypothetical protein Klebsiella pneumoniae
AcrIE8.2
WP 142514135.1 hypothetical protein Klebsiella sp. WCHKI090001
EBR8784912.1 hypothetical protein Salmonella enterica subsp. enterica serovar Agbeni
WP 085843942.1 hypothetical protein Klebsiella pneumoniae
WP 132349276.1 hypothetical protein Klebsiella pneumoniae
hypothetical protein Klebsiella'
WP 038434996.1 hypothetical protein Klebsiella pneumoniae
AcrIE8.1
WP 080850859.1 hypothetical protein Klebsiella pneumoniae
hypothetical protein Klebsiella'
WP 105514343.1 hypothetical protein Cronobacter sakazakii
WP 105626589.1 hypothetical protein Cronobacter malonaticus
WP 097630501.1 hypothetical protein Cronobacter sakazakii
hypothetical protein Cronobacter'
WP 084997554.1 hypothetical protein Cronobacter sakazakii
WP 097585088.1 hypothetical protein Cronobacter sakazakii
WP 085017031.1 hypothetical protein Cronobacter sakazakii
WP 105653527.1 hypothetical protein Cronobacter malonaticus
hypothetical protein Cronobacter'
WP 004386519.1 hypothetical protein Cronobacter sakazakii
hypothetical protein Enterobacteriaceae'
ABU78293.1 hypothetical protein ESA 03064 Cronobacter sakazakii ATCC BAA-894

## b. AcrIF15

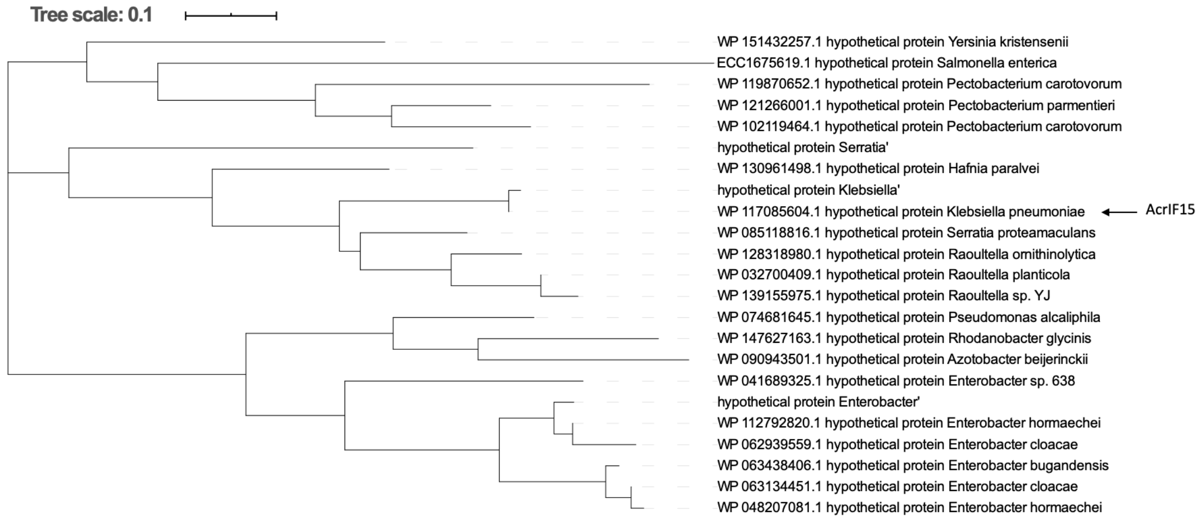
Tree scale: 0.1
WP 151432257.1 hypothetical protein Yersinia kristensenii
ECC1675619.1 hypothetical protein Salmonella enterica
WP 119870652.1 hypothetical protein Pectobacterium carotovorum
WP 121266001.1 hypothetical protein Pectobacterium parmentieri
WP 102119464.1 hypothetical protein Pectobacterium carotovorum
hypothetical protein Serratia'
WP 130961498.1 hypothetical protein Hafnia paralvei
hypothetical protein Klebsiella'
WP 117085604.1 hypothetical protein Klebsiella pneumoniae
AcrIF15
WP 085118816.1 hypothetical protein Serratia proteamaculans
WP 128318980.1 hypothetical protein Raoultella ornithinolytica
WP 032700409.1 hypothetical protein Raoultella planticola
WP 139155975.1 hypothetical protein Raoultella sp. YJ
WP 074681645.1 hypothetical protein Pseudomonas alcaliphila
WP 147627163.1 hypothetical protein Rhodanobacter glycinis
WP 090943501.1 hypothetical protein Azotobacter beijerinckii
WP 041689325.1 hypothetical protein Enterobacter sp. 638
hypothetical protein Enterobacter'
WP 112792820.1 hypothetical protein Enterobacter hormaechei
WP 062939559.1 hypothetical protein Enterobacter cloacae
WP 063438406.1 hypothetical protein Enterobacter bugandensis
WP 063134451.1 hypothetical protein Enterobacter cloacae
WP 048207081.1 hypothetical protein Enterobacter hormaechei

## c. AcrIF16

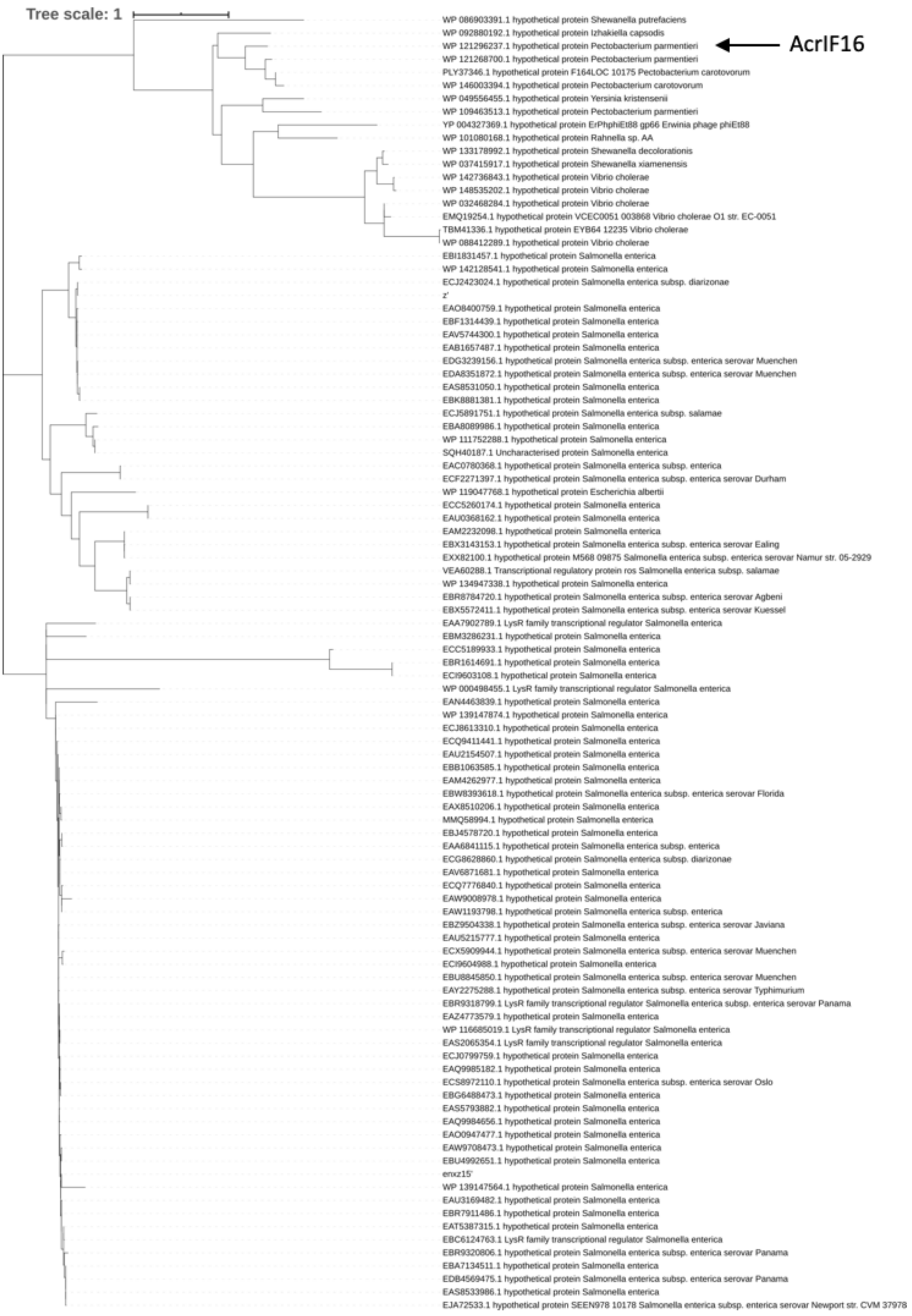

Tree scale: 1
AcrIF16
WP 086903391.1 hypothetical protein Shewanella putrefaciens
WP 092880192.1 hypothetical protein Izhakiella capsodis
WP 121296237.1 hypothetical protein Pectobacterium parmentieri
WP 121268700.1 hypothetical protein Pectobacterium parmentieri
PLY37346.1 hypothetical protein F164LOC 10175 Pectobacterium carotovorum
WP 146003394.1 hypothetical protein Pectobacterium carotovorum
WP 049556455.1 hypothetical protein Yersinia kristensenii
WP 109463513.1 hypothetical protein Pectobacterium parmentieri
YP 004327369.1 hypothetical protein ErPhphiEt88 gp66 Erwinia phage phiEt88
WP 101080168.1 hypothetical protein Rahnella sp. AA
WP 133178992.1 hypothetical protein Shewanella decolorationis
WP 037415917.1 hypothetical protein Shewanella xiamenensis
WP 142736843.1 hypothetical protein Vibrio cholerae
WP 148535202.1 hypothetical protein Vibrio cholerae
WP 032468284.1 hypothetical protein Vibrio cholerae
EMQ19254.1 hypothetical protein VCEC0051 003868 Vibrio cholerae O1 str. EC-0051
TBM41336.1 hypothetical protein EYB64 12235 Vibrio cholerae
WP 088412289.1 hypothetical protein Vibrio cholerae
EBI1831457.1 hypothetical protein Salmonella enterica
WP 142128541.1 hypothetical protein Salmonella enterica
ECJ2423024.1 hypothetical protein Salmonella enterica subsp. diarizonae
z'
EAO8400759.1 hypothetical protein Salmonella enterica
EBF1314439.1 hypothetical protein Salmonella enterica
EAV5744300.1 hypothetical protein Salmonella enterica
EAB1657487.1 hypothetical protein Salmonella enterica
EDG3239156.1 hypothetical protein Salmonella enterica subsp. enterica serovar Muenchen
EDA8351872.1 hypothetical protein Salmonella enterica subsp. enterica serovar Muenchen
EAS8531050.1 hypothetical protein Salmonella enterica
EBK8881381.1 hypothetical protein Salmonella enterica
ECJ5891751.1 hypothetical protein Salmonella enterica subsp. salamae
EBA8089986.1 hypothetical protein Salmonella enterica
WP 111752288.1 hypothetical protein Salmonella enterica
SQH40187.1 Uncharacterised protein Salmonella enterica
EAC0780368.1 hypothetical protein Salmonella enterica subsp. enterica
ECF2271397.1 hypothetical protein Salmonella enterica subsp. enterica serovar Durham
WP 119047768.1 hypothetical protein Escherichia albertii
ECC5260174.1 hypothetical protein Salmonella enterica
EAU0368162.1 hypothetical protein Salmonella enterica
EAM2232098.1 hypothetical protein Salmonella enterica
EBX3143153.1 hypothetical protein Salmonella enterica subsp. enterica serovar Ealing
EXX82100.1 hypothetical protein M568 09875 Salmonella enterica subsp. enterica serovar Namur str. 05-2929
VEA60288.1 Transcriptional regulatory protein ros Salmonella enterica subsp. salamae
WP 134947338.1 hypothetical protein Salmonella enterica
EBR8784720.1 hypothetical protein Salmonella enterica subsp. enterica serovar Agbeni
EBX5572411.1 hypothetical protein Salmonella enterica subsp. enterica serovar Kuessel
EAA7902789.1 LysR family transcriptional regulator Salmonella enterica
EBM3286231.1 hypothetical protein Salmonella enterica
ECC5189933.1 hypothetical protein Salmonella enterica
EBR1614691.1 hypothetical protein Salmonella enterica
ECI9603108.1 hypothetical protein Salmonella enterica
WP 000498455.1 LysR family transcriptional regulator Salmonella enterica
EAN4463839.1 hypothetical protein Salmonella enterica
WP 139147874.1 hypothetical protein Salmonella enterica
ECJ8613310.1 hypothetical protein Salmonella enterica
ECQ9411441.1 hypothetical protein Salmonella enterica
EAU2154507.1 hypothetical protein Salmonella enterica
EBB1063585.1 hypothetical protein Salmonella enterica
EAM4262977.1 hypothetical protein Salmonella enterica
EBW8393618.1 hypothetical protein Salmonella enterica subsp. enterica serovar Florida
EAX8510206.1 hypothetical protein Salmonella enterica
MMQ58994.1 hypothetical protein Salmonella enterica
EBJ4578720.1 hypothetical protein Salmonella enterica
EAA6841115.1 hypothetical protein Salmonella enterica subsp. enterica
ECG8628860.1 hypothetical protein Salmonella enterica subsp. diarizonae
EAV6871681.1 hypothetical protein Salmonella enterica
ECQ7776840.1 hypothetical protein Salmonella enterica
EAW9008978.1 hypothetical protein Salmonella enterica
EAW1193798.1 hypothetical protein Salmonella enterica subsp. enterica
EBZ9504338.1 hypothetical protein Salmonella enterica subsp. enterica serovar Javiana
EAU5215777.1 hypothetical protein Salmonella enterica
ECX5909944.1 hypothetical protein Salmonella enterica subsp. enterica serovar Muenchen
ECI9604988.1 hypothetical protein Salmonella enterica
EBU8845850.1 hypothetical protein Salmonella enterica subsp. enterica serovar Muenchen
EAY2275288.1 hypothetical protein Salmonella enterica subsp. enterica serovar Typhimurium
EBR9318799.1 LysR family transcriptional regulator Salmonella enterica subsp. enterica serovar Panama
EAZ4773579.1 hypothetical protein Salmonella enterica
WP 116685019.1 LysR family transcriptional regulator Salmonella enterica
EAS2065354.1 LysR family transcriptional regulator Salmonella enterica
ECJ0799759.1 hypothetical protein Salmonella enterica
EAQ9985182.1 hypothetical protein Salmonella enterica
ECS8972110.1 hypothetical protein Salmonella enterica subsp. enterica serovar Oslo
EBG6488473.1 hypothetical protein Salmonella enterica
EAS5793882.1 hypothetical protein Salmonella enterica
EAQ9984656.1 hypothetical protein Salmonella enterica
EAO0947477.1 hypothetical protein Salmonella enterica
EAW9708473.1 hypothetical protein Salmonella enterica
EBU4992651.1 hypothetical protein Salmonella enterica
enxz15'
WP 139147564.1 hypothetical protein Salmonella enterica
EAU3169482.1 hypothetical protein Salmonella enterica
EBR7911486.1 hypothetical protein Salmonella enterica
EAT5387315.1 hypothetical protein Salmonella enterica
EBC6124763.1 LysR family transcriptional regulator Salmonella enterica
EBR9320806.1 hypothetical protein Salmonella enterica subsp. enterica serovar Panama
EBA7134511.1 hypothetical protein Salmonella enterica
EDB4569475.1 hypothetical protein Salmonella enterica subsp. enterica serovar Panama
EAS8533986.1 hypothetical protein Salmonella enterica
EJA72533.1 hypothetical protein SEEN978 10178 Salmonella enterica subsp. enterica serovar Newport str. CVM 37978

## d. AcrIF17

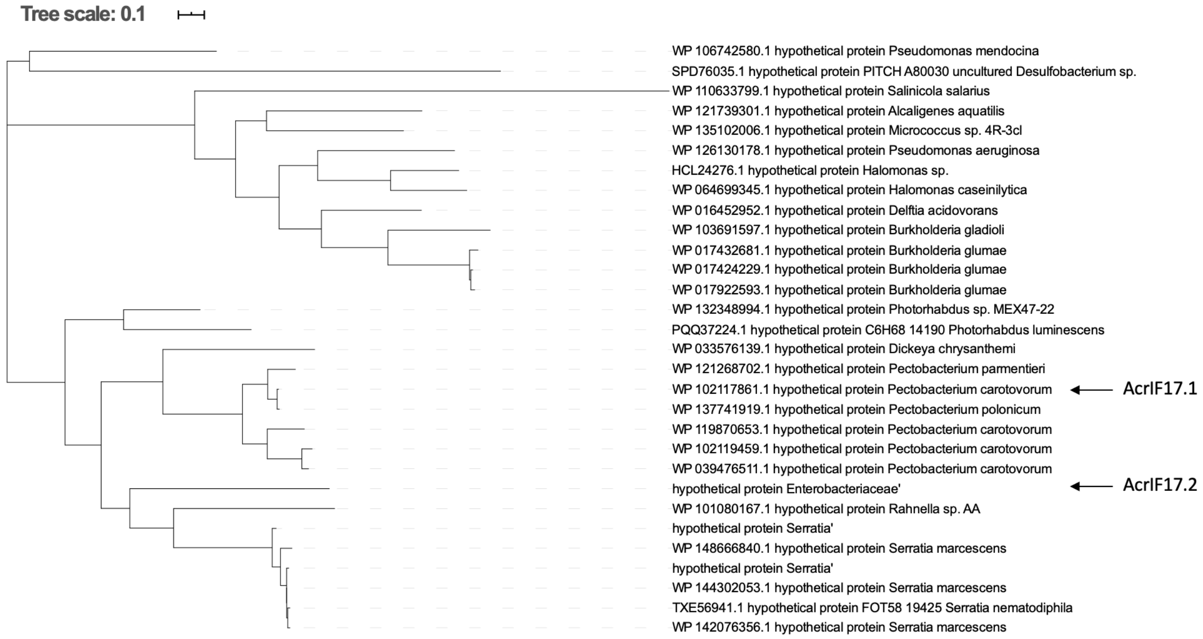


## e. AcrIF18*

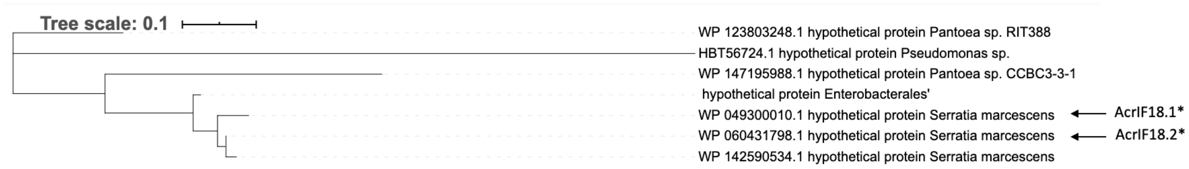


## f. AcrIF19

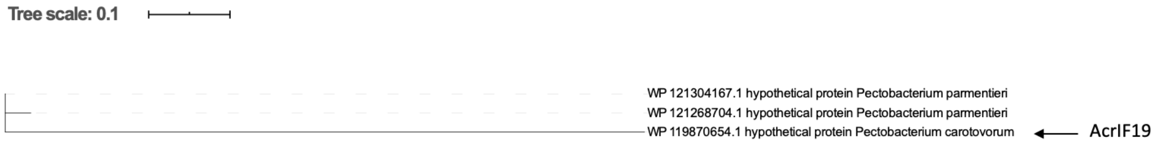

## g. AcrIF20

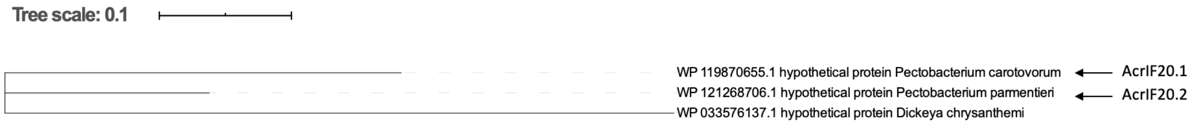


## h. AcrIF21

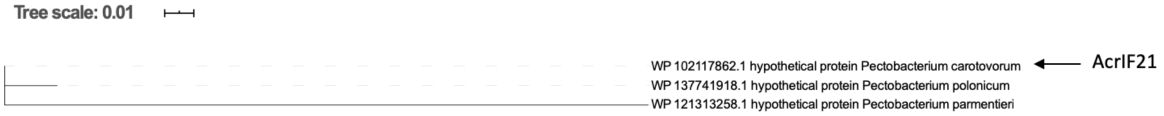


## i. AcrIF22*

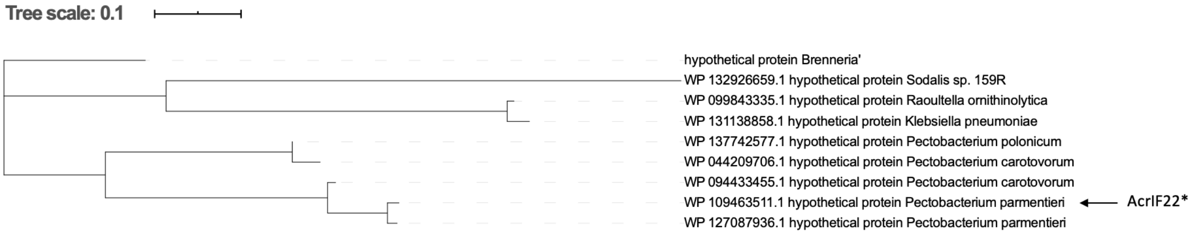

## j. AcrIF23

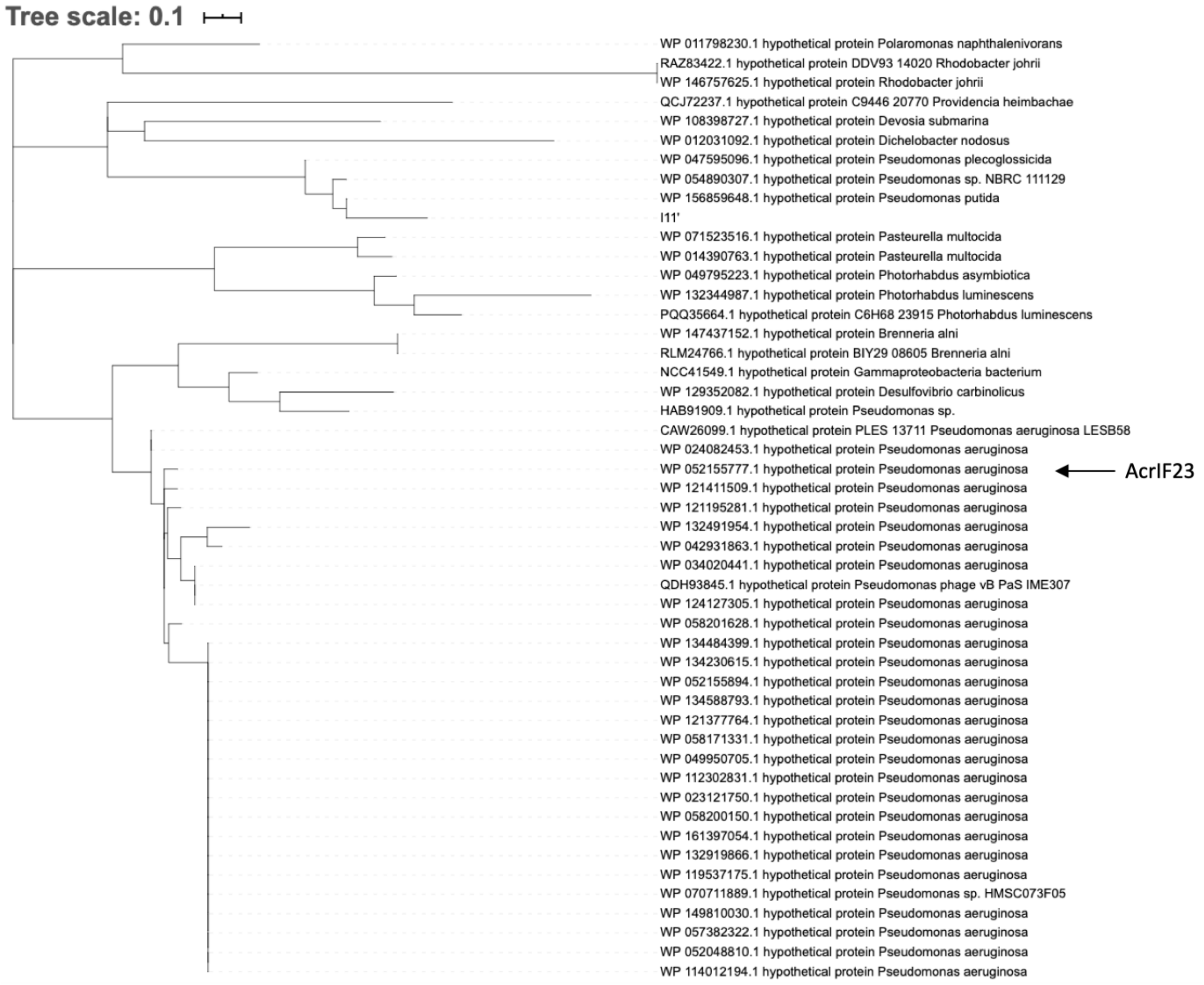
Tree scale: 0.1
WP 011798230.1 hypothetical protein Polaromonas naphthalenivorans
RAZ83422.1 hypothetical protein DDV93 14020 Rhodobacter johrii
WP 146757625.1 hypothetical protein Rhodobacter johrii
QCJ72237.1 hypothetical protein C9446 20770 Providencia heimbachae
WP 108398727.1 hypothetical protein Devosia submarina
WP 012031092.1 hypothetical protein Dichelobacter nodosus
WP 047595096.1 hypothetical protein Pseudomonas plecoglossicida
WP 054890307.1 hypothetical protein Pseudomonas sp. NBRC 111129
WP 156859648.1 hypothetical protein Pseudomonas putida
I11'
WP 071523516.1 hypothetical protein Pasteurella multocida
WP 014390763.1 hypothetical protein Pasteurella multocida
WP 049795223.1 hypothetical protein Photorhabdus asymbiotica
WP 132344987.1 hypothetical protein Photorhabdus luminescens
PQQ35664.1 hypothetical protein C6H68 23915 Photorhabdus luminescens
WP 147437152.1 hypothetical protein Brenneria alni
RLM24766.1 hypothetical protein BIY29 08605 Brenneria alni
NCC41549.1 hypothetical protein Gammaproteobacteria bacterium
WP 129352082.1 hypothetical protein Desulfovibrio carbinolicus
HAB91909.1 hypothetical protein Pseudomonas sp.
CAW26099.1 hypothetical protein PLES 13711 Pseudomonas aeruginosa LESB58
WP 024082453.1 hypothetical protein Pseudomonas aeruginosa
WP 052155777.1 hypothetical protein Pseudomonas aeruginosa
AcrIF23
WP 121411509.1 hypothetical protein Pseudomonas aeruginosa
WP 121195281.1 hypothetical protein Pseudomonas aeruginosa
WP 132491954.1 hypothetical protein Pseudomonas aeruginosa
WP 042931863.1 hypothetical protein Pseudomonas aeruginosa
WP 034020441.1 hypothetical protein Pseudomonas aeruginosa
QDH93845.1 hypothetical protein Pseudomonas phage vB PaS IME307
WP 124127305.1 hypothetical protein Pseudomonas aeruginosa
WP 058201628.1 hypothetical protein Pseudomonas aeruginosa
WP 134484399.1 hypothetical protein Pseudomonas aeruginosa
WP 134230615.1 hypothetical protein Pseudomonas aeruginosa
WP 052155894.1 hypothetical protein Pseudomonas aeruginosa
WP 134588793.1 hypothetical protein Pseudomonas aeruginosa
WP 121377764.1 hypothetical protein Pseudomonas aeruginosa
WP 058171331.1 hypothetical protein Pseudomonas aeruginosa
WP 049950705.1 hypothetical protein Pseudomonas aeruginosa
WP 112302831.1 hypothetical protein Pseudomonas aeruginosa
WP 023121750.1 hypothetical protein Pseudomonas aeruginosa
WP 058200150.1 hypothetical protein Pseudomonas aeruginosa
WP 161397054.1 hypothetical protein Pseudomonas aeruginosa
WP 132919866.1 hypothetical protein Pseudomonas aeruginosa
WP 119537175.1 hypothetical protein Pseudomonas aeruginosa
WP 070711889.1 hypothetical protein Pseudomonas sp. HMSC073F05
WP 149810030.1 hypothetical protein Pseudomonas aeruginosa
WP 057382322.1 hypothetical protein Pseudomonas aeruginosa
WP 052048810.1 hypothetical protein Pseudomonas aeruginosa
WP 114012194.1 hypothetical protein Pseudomonas aeruginosa

### k. AcrIF24

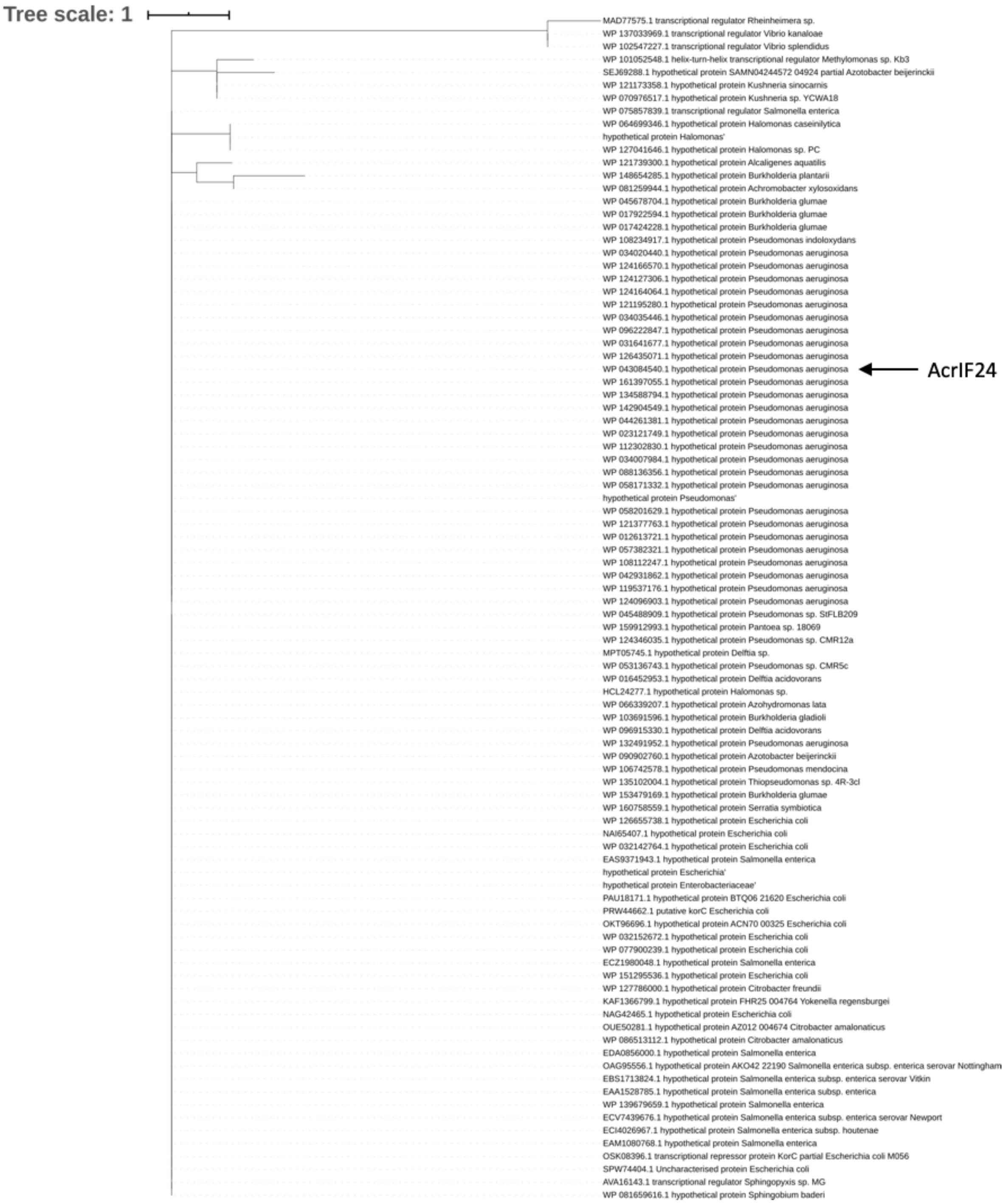


**Supplementary Fig. 3. Distribution of the identified Acrs across bacterial taxa.** For each Acr family, phylogenetic analyses were performed with the homologs retrieved after PSI-BLAST searches (up to three iterations, only considering hits with e-values <$10^{-4}$ for PSSM generation). Multiple alignments were performed with the MUSCLE software and Maximum Likelihood

phylogenetic trees were constructed using MEGA. Branches are labeled with the corresponding protein accession number, followed by the name of the bacterial host species. Arrows indicate the positions in the tree where the Acr homologs tested in this study are found.

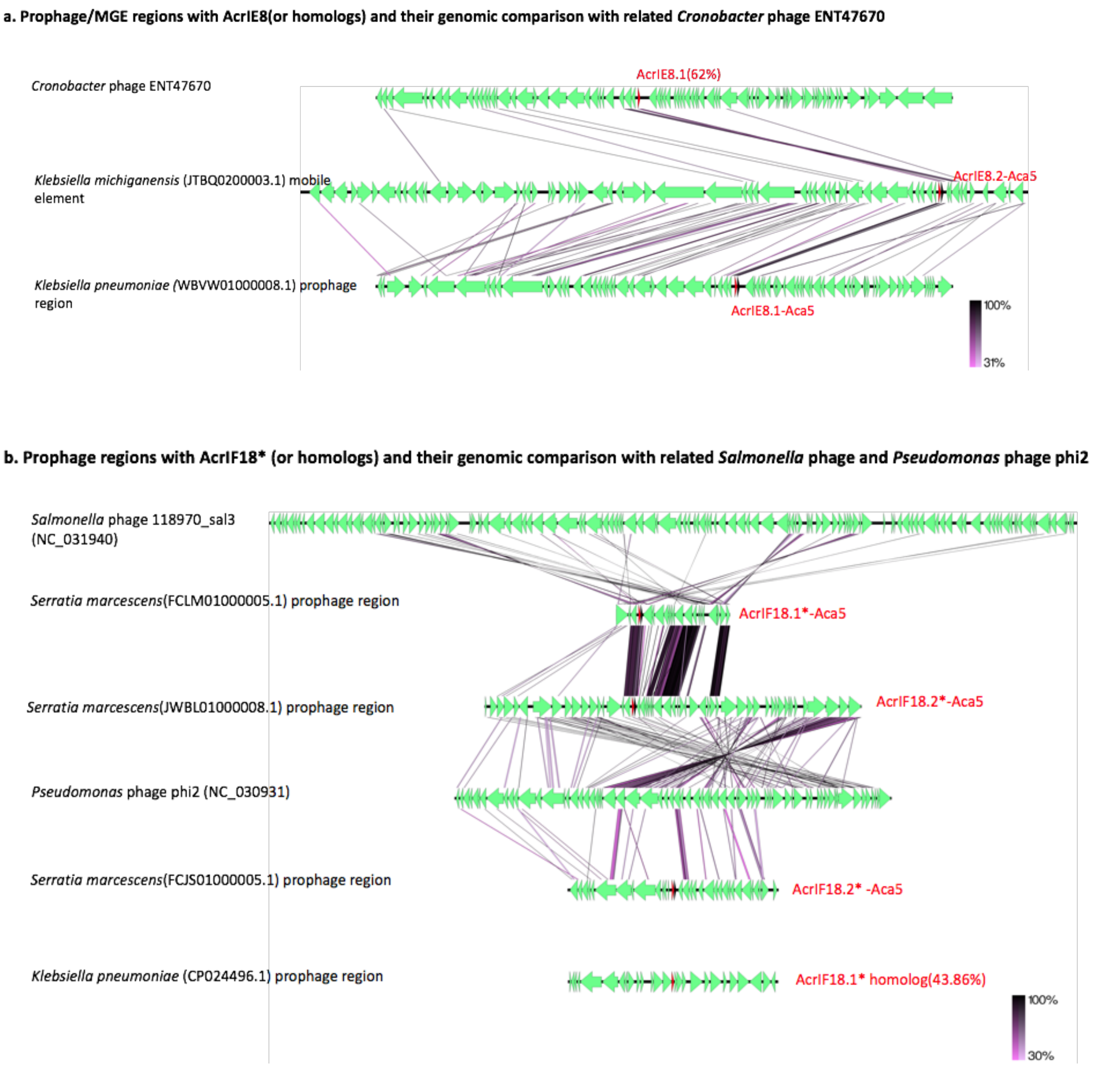

**c. Prophage regions with AcrIF16,AcrIF17,AcrIF19,AcrIF20,AcrIF21 (or homologs) and their genomic comparison with related *Enterobacter* phage mEP237,mEP460 and *Pectobacterium* phage ZF40**

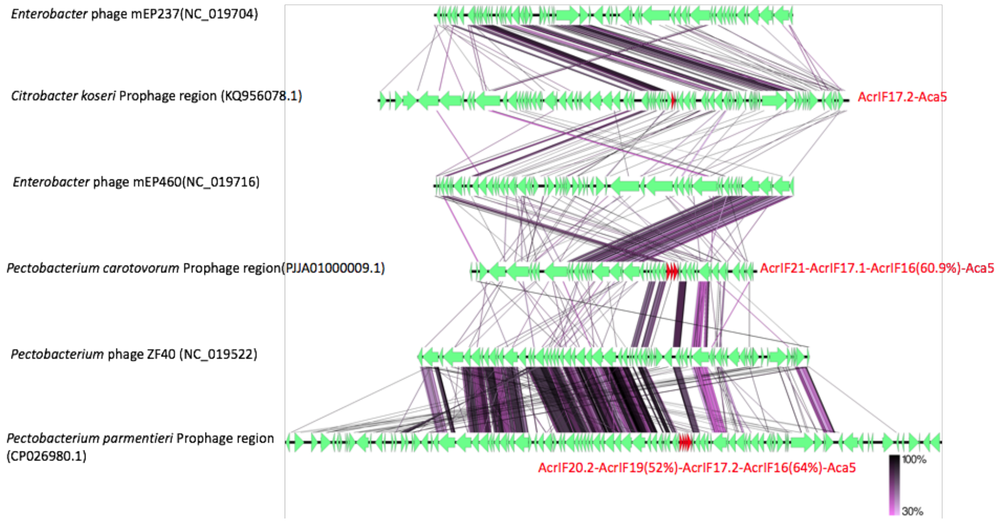


**Supplementary Fig. 4. Genome comparison among prophages/MGEs having anti-CRISPRs with related phages:** Tblastx has been performed to compare different prophage genomes with their closely related phages (PHASTER best hit). The genomic regions are visualized with EasyFig V2.2.2. All encoded proteins except representative anti-CRISPRs (red) are coloured green. Sequence similarities are shown with gradient (purple to black; between 30% and 100%) straight lines. **(a)** The prophages/MGEs with AcrIE8 share sequence similarity with *Cronobacter* phage ENT47670 genome. All of these genomes have AcrIE8 but the position of *acrs*, neighbouring gene cassettes are variable among different genomes. **(b)** The prophages carrying AcrIF18* share sequence similarity with *Salmonella* phage 118970_sal3 and *Pseudomonas* phage phi2.These phage genomes do not have AcrIF18* and the position of *acrs*, neighbouring gene cassettes are variable among different prophage genomes. **(c)** The anti-CRISPRs AcrIF16, AcrIF17, AcrIF19, AcrIF20, AcrIF21 and the homologs of these Acrs are mostly found to be clustered in the same prophage regions. These regions have sequence similarity with *Enterobacter* phage mEP460, mEP237, and *Pectobacterium* phage ZF40. The phage genomes do not have these anti-CRISPRs, and the position of *acrs*, neighbouring gene cassettes are variable among different prophage genomes. A detailed analysis with *Pectobacterium* phage ZF40 is described in the text and Figure 3.

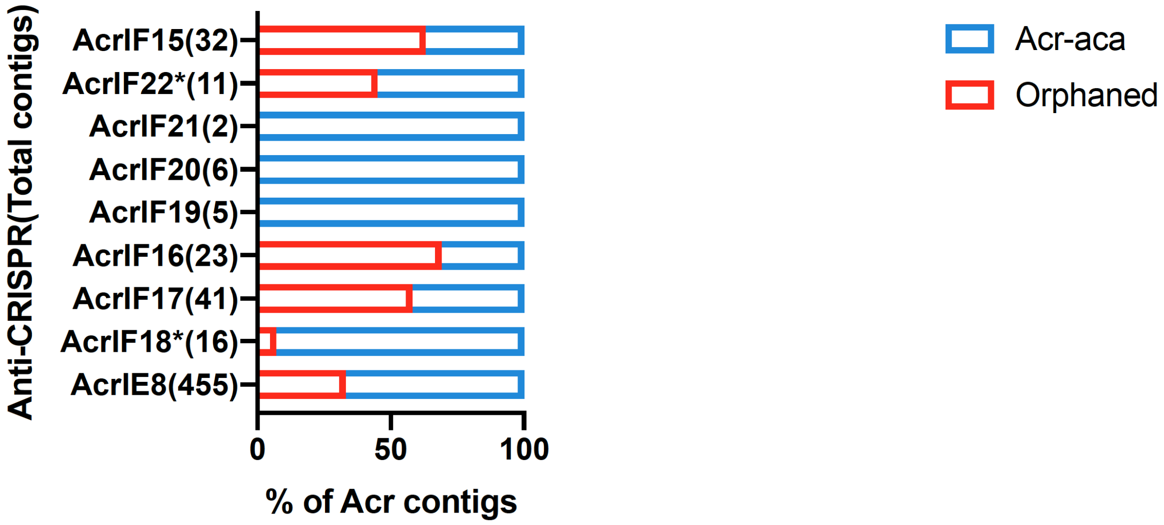


**Supplementary Fig. 5. Proportion of newly identified acrs associated with a known *aca* gene.** Homology search of the different acrs presented in this work against all Refseq bacterial contigs (total n = 18654021) revealed a significant number of orphaned Acrs in the bacterial genomes.

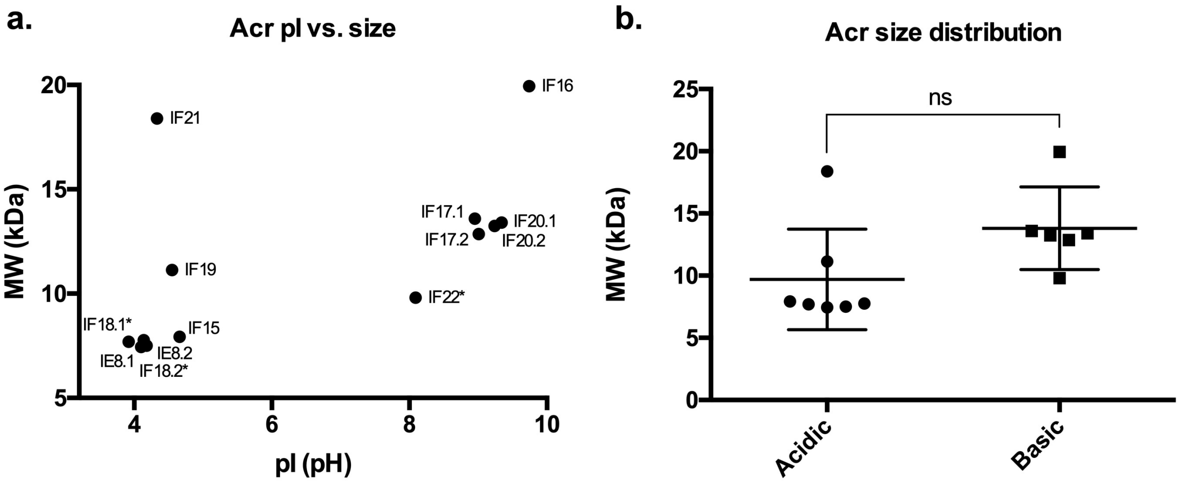


**Supplementary Fig. 6.** Analysis of the molecular weights (MW) and isoelectric points (pI) of the newly identified I-F and I-E Acrs. **a.** Scatterplot showing the relationship between Acr pI and MW. **b.** Acr distribution for Acrs grouped by acidic (pH < 7) and basic (pH > 7) pIs. No statistical significance (ns, p > 0.05) was observed between the basic and acidic Acr groups (unpaired t-test; p value = 0.0733). Asterisk denotes that the Arc in question is a dual I-F and I-E inhibitor. Data are presented

as mean ± SEM of n = 7 (acidic) and n=6 (basic) independent samples. Source data are available in the Source Data file.

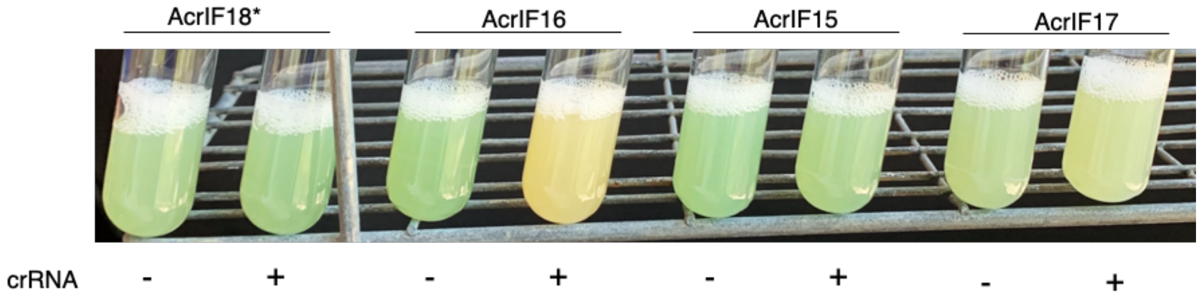


**Supplementary Fig. 7. CRISPRi-based repression of pyocyanin production.** Assay results showing a representative picture of the observed change in color observed for the expression of AcrIF18*, AcrIF16, AcrIF15 and AcrIF17, in the presence (+) or absence (-) of a crRNA targeting the *phzM* gene, in a delta Cas3 PA14 genomic background.

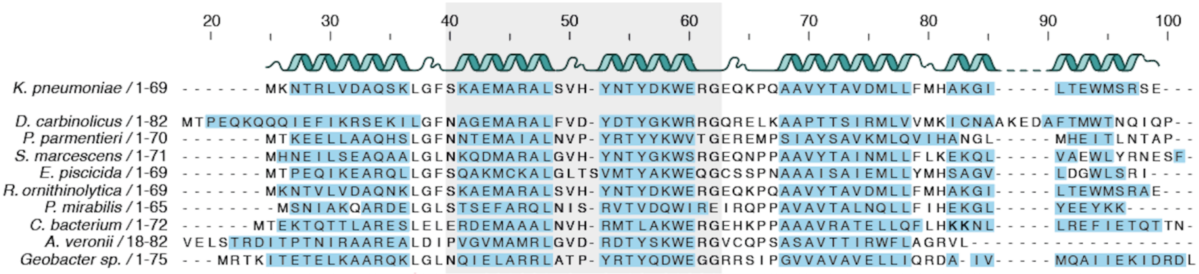


**Supplementary Fig. 8.** Secondary structure prediction for the diverse Aca9 homologs displayed in the alignment form Figure 4b. Predicted secondary structure alpha-helix regions are illustrated as ribbons for the *K. pneumoniae* homolog (top) and blue shadings indicate the corresponding predictions across the different orthologs. The predicted HTH DNA binding domain is highlighted with a gray shaded area.

**Aca9 (QBI37412.1)**

| # | PDB/Pfam Hit | Prob. | E-value | Query HMM |
|---|---|---|---|---|
| 1 | 5J2Y_A; Quorum-sensing repressor | 99,6 | 4,7E-18 | 2-62 |
| 2 | 4O8B_A; HTH motif, transcriptional activator | 99,6 | 6,7E-17 | 1-61 |
| 3 | 2P5T_E; PezA helix-turn-helix motif | 99,5 | 1,4E-16 | 1-63 |
| 4 | 2EWT_A; DNA-binding domain of BldD | 99,5 | 2,4E-15 | 2-62 |
| 5 | 3IVP_A; transposon-related DNA-binding | 99,4 | 2,1E-15 | 1-60 |
| 6 | PF15731.5; MqsA_antitoxin | 99,4 | 2.4E-15 | 2-62 |
| 7 | 6H49_A; SaPI, Repressor | 99,4 | 4,6E-15 | 2-63 |
| 8 | 3U3W_A; HTH DNA-binding, Transcriptional activator | 99,4 | 6,0E-15 | 2-64 |
| 9 | 2EBY_A; HTH-type transcriptional regulator ybaQ | 99,4 | 1,5E-14 | 2-67 |
| 10 | 4I6R_B; helix-turn-helix, transcriptional regulator | 99,4 | 2,7E-14 | 2-63 |

**AcrIF24 (WP_043084540.1)**

| # | PDB/Pfam Hit | Prob. | E-value | Query HMM |
|---|---|---|---|---|
| 1 | 2K9Q_B; helix-turn-helix | 94,1 | 0,1 | 164-225 |
| 2 | 3IVP_D; transposon-related DNA-binding | 93,7 | 0,12 | 164-225 |
| 3 | 3OMT_B; uncharacterized protein | 93,4 | 0,2 | 164-225 |
| 4 | 2EWT_A; putative DNA-binding protein | 93,3 | 0,21 | 164-225 |
| 5 | 2EBY_B; Putative HTH-type transcriptional regulator | 93,0 | 0,23 | 161-225 |
| 6 | PF15731.5; Antitoxin component | 92,8 | 0,16 | 163-225 |
| 7 | PF14549.6; DNA-binding transcriptional regulator Cro | 92,8 | 0,21 | 167-225 |
| 8 | PF08965.10; DUF1870 Domain | 92,6 | 0,21 | 163-224 |
| 9 | 2R1J_R; Helix-turn-helix Repressor protein C2/DN | 92,6 | 0,41 | 164-225 |
| 10 | 1X57_A; helix-turn-helix EDF1 | 92,3 | 0,39 | 164-225 |

**Supplementary Fig. 9.** Summary of the HHPred analyses performed for Aca9 and AcrIF24. The top ten PDB/Pfam protein hits and associated domains ranked according to the HHPred probability score are listed, together with the corresponding e-values and matching amino acid positions in the protein query (query HMM).

## References:


1. Thoma, S. & Schobert, M. An improved Escherichia coli donor strain for diparental mating. *FEMS Microbiol. Lett.* **294**, 127–132 (2009).
2. Pawluk, A. *et al.* Inactivation of CRISPR-Cas systems by anti-CRISPR proteins in diverse bacterial species. *Nat Microbiol* **1**, 16085 (2016).

3. Marino, N. D. *et al.* Discovery of widespread type I and type V CRISPR-Cas inhibitors. *Science* **362**, 240–242 (2018).

4. Cady, K. C., Bondy-Denomy, J., Heussler, G. E., Davidson, A. R. & O'Toole, G. A. The CRISPR/Cas adaptive immune system of Pseudomonas aeruginosa mediates resistance to naturally occurring and engineered phages. *J. Bacteriol.* **194**, 5728–5738 (2012).

5. Blower, T. R., Short, F. L., Fineran, P. C. & Salmond, G. P. C. Viral molecular mimicry circumvents abortive infection and suppresses bacterial suicide to make hosts permissive for replication. *Bacteriophage* **2**, 234–238 (2012).

6. Jackson, S. A., Birkholz, N., Malone, L. M. & Fineran, P. C. Imprecise Spacer Acquisition Generates CRISPR-Cas Immune Diversity through Primed Adaptation. *Cell Host Microbe* **25**, 250–260.e4 (2019).

7. Richter, C., Gristwood, T., Clulow, J. S. & Fineran, P. C. In vivo protein interactions and complex formation in the Pectobacterium atrosepticum subtype I-F CRISPR/Cas System. *PLoS One* **7**, e49549 (2012).

8. Bondy-Denomy, J. *et al.* Multiple mechanisms for CRISPR-Cas inhibition by anti-CRISPR proteins. *Nature* **526**, 136–139 (2015).

9. Couvin, D. *et al.* CRISPRCasFinder, an update of CRISRFinder, includes a portable version, enhanced performance and integrates search for Cas proteins. *Nucleic Acids Res.* **46**, W246–W251 (2018).

10. Kumar, S., Stecher, G., Li, M., Knyaz, C. & Tamura, K. MEGA X: Molecular Evolutionary Genetics Analysis across Computing Platforms. *Molecular Biology and Evolution* vol. 35 1547–1549 (2018).

11. Biswas, A., Gagnon, J. N., Brouns, S. J. J., Fineran, P. C. & Brown, C. M. CRISPRTarget: bioinformatic prediction and analysis of crRNA targets. *RNA Biol.* **10**, 817–827 (2013).

12. Letunic, I. & Bork, P. Interactive Tree Of Life (iTOL) v4: recent updates and new developments. *Nucleic Acids Res.* **47**, W256–W259 (2019).

13. Söding, J., Biegert, A. & Lupas, A. N. The HHpred interactive server for protein homology detection and structure prediction. *Nucleic Acids Res.* **33**, W244–8 (2005).

14. Kelley, L. A., Mezulis, S., Yates, C. M., Wass, M. N. & Sternberg, M. J. E. The Phyre2 web portal for protein modeling, prediction and analysis. *Nat. Protoc.* **10**, 845–858 (2015).
15. Potter, S. C. *et al.* HMMER web server: 2018 update. *Nucleic Acids Res.* **46**, W200–W204 (2018).
16. Sullivan, M. J., Petty, N. K. & Beatson, S. A. Easyfig: a genome comparison visualizer. *Bioinformatics* **27**, 1009–1010 (2011).
17. Paez-Espino, D. *et al.* IMG/VR: a database of cultured and uncultured DNA Viruses and retroviruses. *Nucleic Acids Res.* **45**, D457–D465 (2017).
18. Russel, J., Pinilla-Redondo, R., Mayo-Muñoz, D., Shah, S. A. & Sørensen, S. J. CRISPRCasTyper: An automated tool for the identification, annotation and classification of CRISPR-Cas loci. doi:10.1101/2020.05.15.097824.
19. Drozdetskiy, A., Cole, C., Procter, J. & Barton, G. J. JPred4: a protein secondary structure prediction server. *Nucleic Acids Res.* **43**, W389–94 (2015).
