# Supplementary material for: Discovery of multiple anti-CRISPRs highlights anti-defense gene clustering in mobile genetic elements
Source: Nat Commun. 2020 Nov 6;11:5652. doi: 10.1038/s41467-020-19415-3 (PMC7648647; doi:10.1038/s41467-020-19415-3)
Supplement: Supplementary file 2 — Description of Additional Supplementary Files [file 41467_2020_19415_MOESM2_ESM.pdf]

Description of Additional Supplementary Files

File Name: Supplementary Data 1
Description: *Information summary for the identified Acrs. Information includes: the accession numbers for the different Acrs (and homologs) tested in this work, predicted isoelectric points (pI) for the proteins, amino acid (aa) sequence lengths, molecular weights (MW), positions in the genomes of the bacteria where acrs are found, positions and (sub)types of detected CRISPR arrays and Cas operons in the bacterial host genomes, presence/absence of self-targeting within the host genomes, positions of the identified prophage regions found within the genomes of bacteria encoding a validated Acrs.*

File Name: Supplementary Data 2
Description: *List and analysis of self-targeting spacers. The following information is provided: the spacer number that self-targeting spacers occupy in the array of origin and corresponding CRISPR-Cas subtype, the genomic position (bp) where self-targeting occurs, the sequence of the self-targeting spacer, number of mismatches, predicted PAMs, whether a phage is predicted within the predicted self-targeted region, the ORF and predicted protein-coding genes targeted (when possible).*

File Name: Supplementary Data 3
Description: *Summary of the CRISPR-Cas sequence identity comparisons. The results from BLATSp comparisons between the Cas orthologs of the different model CRISPR-Cas systems tested and the selected endogenous CRISPR-Cas systems (hosts from which the selected acrs originate) is shown. An average score of the percentage sequence identities was calculated for all one-to-one comparisons.*

File Name: Supplementary Data 4
Description: *Analysis of the genomic contexts and MGE origins of the homologs of the validated Acrs. The datasheet includes a list of accession numbers and protein sequences for all identified homologs of AcrIE8, AcrIF15, AcrIF16, AcrIF17, AcrIF18*, AcrIF19, AcrIF16, AcrIF20, AcrIF21, AcrIF22* that were used to build the phylogenetic trees of the Acr families. MGE-related genes used to determine the genetic context of each Acr homolog are listed. A summary count of the homologs found in phage, plasmid, and unknown genomic contexts is provided for each Acr.*

File Name: Supplementary Data 5
Description: *Summary of proteins search results for phage genome regions represented. Annotations were obtained performing searches against the pfam and Prokaryotic Virus Orthologous group (pVOG) databases.*

File Name: Supplementary Data 6
Description: *List of Aca9 homologs used in this work. MGE-related genes used to determine the genetic context of each homolog are listed.*

File Name: Supplementary Data 7
Description: *Lists of accession numbers and protein sequences for the homologs of AcrIF23 and AcrIF24 used in this work.*

File Name: Supplementary Data 8
Description: *List of putative antibiotic resistance genes identified on the Klebsiella pneumoniae ABFQB plasmid unitig_1 (CP036439.1). BLAST searches were performed against the Comprehensive*

Antibiotic Resistance Database (CARD). Positions for the location of genes in the plasmid genome, the e-value for the matches, and a short description of the gene function are indicated.

File Name: Supplementary Data 9
Description: *Lists of accession numbers and protein sequences for the homologs of Aca5 used in this work.*
