# Supplementary material for: Discovery of multiple anti-CRISPRs highlights anti-defense gene clustering in mobile genetic elements
Source: Nat Commun. 2020 Nov 6;11:5652. doi: 10.1038/s41467-020-19415-3 (PMC7648647; doi:10.1038/s41467-020-19415-3)
Supplement: Supplementary file 12 — Reporting Summary [file 41467_2020_19415_MOESM12_ESM.pdf]

nature research

Corresponding author(s): Joseph Bondy-Denomy & Peter Fineran

Last updated by author(s): 2020/09/15

Reporting Summary

Nature Research wishes to improve the reproducibility of the work that we publish. This form provides structure for consistency and transparency in reporting. For further information on Nature Research policies, see our Editorial Policies and the Editorial Policy Checklist.

Statistics

For all statistical analyses, confirm that the following items are present in the figure legend, table legend, main text, or Methods section.

n/a	Confirmed	
☐	☒	The exact sample size (*n*) for each experimental group/condition, given as a discrete number and unit of measurement
☐	☒	A statement on whether measurements were taken from distinct samples or whether the same sample was measured repeatedly
☐	☒	The statistical test(s) used AND whether they are one- or two-sided<br>*Only common tests should be described solely by name; describe more complex techniques in the Methods section.*
☒	☐	A description of all covariates tested
☒	☐	A description of any assumptions or corrections, such as tests of normality and adjustment for multiple comparisons
☐	☒	A full description of the statistical parameters including central tendency (e.g. means) or other basic estimates (e.g. regression coefficient) AND variation (e.g. standard deviation) or associated estimates of uncertainty (e.g. confidence intervals)
☒	☐	For null hypothesis testing, the test statistic (e.g. *F*, *t*, *r*) with confidence intervals, effect sizes, degrees of freedom and *P* value noted<br>*Give P values as exact values whenever suitable.*
☒	☐	For Bayesian analysis, information on the choice of priors and Markov chain Monte Carlo settings
☒	☐	For hierarchical and complex designs, identification of the appropriate level for tests and full reporting of outcomes
☒	☐	Estimates of effect sizes (e.g. Cohen's *d*, Pearson's *r*), indicating how they were calculated

Our web collection on statistics for biologists contains articles on many of the points above.

Software and code

Policy information about availability of computer code

Data collection	No software was used for data collection.
Data analysis	Numerical data were analyzed and plotted with GraphPad Prism 6.0 Software. HHPred (February 2020) searches were carried out for the prediction of protein domains. Protein secondary structure predictions were carried out by JPred4 and Phyre2. Detection of antibiotic resistance genes was performed via BLAST (against the Comprehensive Antibiotic Resistance Database (CARD) (February 2020). CRISPRtarget (January 2020) was used to identify selt-targeting hits. CRISPRCasFinder v4.2.17 and CRISPRCasTyper v1.1.5 were used to predict cas operons and CRISPR arrays. IMG/VR (March 2020) was scanned to identify anti-defense gene homologs. Adobe illustrator v24.0.2 was employed to make figures and diagrams. iTOL v5.6.3 was employed to edit phylogenetic trees. Protein sequence alignments and phylogenetic trees were performed in MEGAX (including MUSCLE v3.8.1551 for protein alignments). HMMER3.0 was used to make protein HMM profiles. Easyfig v2.2.2. was used to perform genome comparisons.

For manuscripts utilizing custom algorithms or software that are central to the research but not yet described in published literature, software must be made available to editors and reviewers. We strongly encourage code deposition in a community repository (e.g. GitHub). See the Nature Research guidelines for submitting code & software for further information.

Data

Policy information about availability of data

All manuscripts must include a data availability statement. This statement should provide the following information, where applicable:

- Accession codes, unique identifiers, or web links for publicly available datasets
- A list of figures that have associated raw data
- A description of any restrictions on data availability

All data is available in the Supplementary Data files or Source Data files. Figures with associated raw data are: 1a, 1b, 1c, 2b, 2c, 2d, 3, 4b, 5c, 6a.

Public datasets:
Comprehensive Antibiotic Resistance Database (CARD): https://card.mcmaster.ca/
NCBI-NR: https://www.ncbi.nlm.nih.gov/refseq/about/nonredundantproteins/
IMG/VR: https://img.jgi.doe.gov/cgi-bin/vr/main.cgi

Field-specific reporting

Please select the one below that is the best fit for your research. If you are not sure, read the appropriate sections before making your selection.

[x] Life sciences   [ ] Behavioural & social sciences   [ ] Ecological, evolutionary & environmental sciences

For a reference copy of the document with all sections, see nature.com/documents/nr-reporting-summary-flat.pdf

Life sciences study design

All studies must disclose on these points even when the disclosure is negative.

Sample size	Three random colonies (exception: two in results displayed in Fig. 5c) were picked as biological replication for each assay. No sample-size calculations were performed. The sample sizes were judged to be sufficient based on the reproducibility of measurements between groups and are in line with the sample sizes used in the same type of experiments carried out in this field.
Data exclusions	No data was excluded.
Replication	Values in the figures represent the average of independent replicates stemming from separate colonies. We observed data is reproducible across independent experiments.
Randomization	Random colonies of transformants were picked as biological replicates.
Blinding	Blinding is not relevant to the study because no group allocation occurs.

Reporting for specific materials, systems and methods

We require information from authors about some types of materials, experimental systems and methods used in many studies. Here, indicate whether each material, system or method listed is relevant to your study. If you are not sure if a list item applies to your research, read the appropriate section before selecting a response.

Materials & experimental systems

n/a	Involved in the study	
[x]	[ ]	Antibodies
[x]	[ ]	Eukaryotic cell lines
[x]	[ ]	Palaeontology and archaeology
[x]	[ ]	Animals and other organisms
[x]	[ ]	Human research participants
[x]	[ ]	Clinical data
[x]	[ ]	Dual use research of concern

Methods

n/a	Involved in the study	
[x]	[ ]	ChIP-seq
[x]	[ ]	Flow cytometry
[x]	[ ]	MRI-based neuroimaging
